# ATM inhibition enhance immunotherapy by activating STING signaling and augmenting MHC Class I

**DOI:** 10.1038/s41419-024-06911-3

**Published:** 2024-07-20

**Authors:** Chunya Li, Boyu Wang, Jingyao Tu, Chaofan Liu, Yuan Wang, Junjie Chen, Yongbiao Huang, Bo Liu, Xianglin Yuan

**Affiliations:** 1 Department of Oncology, Tongji Hospital, Tongji Medical College, Huazhong University of Science and Technology, Wuhan, China; 2 Department of Thoracic Surgery, Tongji Hospital, Tongji Medical College, Huazhong University of Science and Technology, Wuhan, China

**Keywords:** Cancer immunotherapy, Cancer microenvironment

## Abstract

Accumulating evidence supports the concept that DNA damage response targeted therapies can improve antitumor immune response by increasing the immunogenicity of tumor cells and improving the tumor immune microenvironment. Ataxia telangiectasia mutated (ATM) is a core component of the DNA repair system. Although the ATM gene has a significant mutation rate in many human cancers, including colorectal, prostate, lung, and breast, it remains understudied compared with other DDR-involved molecules such as PARP and ATR. Here, we found that either gene knockout or drug intervention, ATM inhibition activated the cGAS/STING pathway and augmented MHC class I in CRC cells, and these effects could be amplified by radiation. Furthermore, we found that MHC class I upregulation induced by ATM inhibition is dependent on the activation of the NFκB/IRF1/NLRC5 pathway and independent of STING. Animal experiments have shown increasing infiltration and cytotoxic function of T cells and better survival in ATM-deficient tumors. This work indicated that ATM nonsense mutation predicted the clinical benefits of radiotherapy combined with immune checkpoint blockade for patients with CRC. It also provides a molecular mechanism rationale for ATM-targeted agents for patients with CRC.

## Introduction

As a revolutionary and influential treatment, immunotherapy has obtained durable clinical responses, but only a subset of patients can benefit from it. According to Global Cancer Statistics 2020, colorectal cancer (CRC), as the third-highest incidence and second-highest mortality rate of cancer, seriously endangers human health [1]. Except for ~15% of CRC patients with high microsatellite instability or deficient mismatch repair (dMMR) may be sensitive to immunotherapy [2–4], most CRC patients do not benefit from immunotherapy. Strategies for overcoming intrinsic and acquired resistance to immunotherapy are urgently needed.

On the one hand, the tumor immune microenvironment (TME) of microsatellite stable CRCs contains low TMB and lacks expression of inflammatory gene signatures [5, 6]. The lack of a detectable immune response may reflect inefficiencies in antigen presentation and initiating adaptive immune response. Therefore, the tumor may have defects associated with APCs and T-cell infiltration into the tumor tissue. On the other hand, tumor cell autonomic factors, including oncogenic signaling pathways, mutational load, PD-L1 expression and downregulation of major histocompatibility complex (MHC) class I, inhibit the therapeutic of immunotherapy by modulating the TME [7, 8]. Developing predictive biomarkers and novel therapeutic strategies to improve the TME is a vital measure to overcome tumor immune resistance.

More and more studies have shown that DNA-sensing pathways activated by DNA damage attract innate immune cells into tumors and subsequently activate adaptive response [9, 10]. Cytoplasmic DNA and micronucleus generated by DNA damage can be recognized by cGAMP synthase (cGAS), thus binding and activating STING protein to induce interferon [11–13], mimicking viral infection. Radiotherapy and DDR inhibitors activate the cytoplasmic nucleic acid sensing pathway, and propagation of the resulting inflammatory signal reshapes the tumor immune microenvironment and improves immunotherapy efficacy. Radiotherapy combined with DDR-targeted therapy has been studied for its potential to transform a ‘cold’ tumor into a ‘hot’ tumor [14], but studies considering immunotherapy are still lacking in colorectal tumors.

It has been reported that the prevalence of somatic DDR defects in CRC is as high as 10–30% [15–18], and the prevalence of ATM mutation is 7% in non-hypermutational tumors of consensus molecular subtypes-3 [19]. ATM can initiate a cascade for the DSB signaling response as the master regulator of DSB response, resulting in the phosphorylation of lots of substrates when cells are undergoing the DDR [20], thus promoting cell survival at low toxicity and apoptosis at high toxicity. Some studies have shown that cancer patients with ATM mutations have benefited from ICB therapy compared to those without ATM mutations [21–24], as ATM mutations were found to be correlated with TMB-H, increased expression of PD-L1 and ISGs [22, 25, 26]. Other studies have shown that ATM mutations or loss of ATM protein might be associated with adverse prognostic effects [27–32]. One multicenter study of 227 clinical samples found that ATM mutations were independently associated with longer survival in patients with metastatic colorectal cancer (mCRC) [33]. Only one case report study reported that two microsatellite stable colorectal cancer (MSS CRC) patients with ATM mutations benefited from anti-PD-1 checkpoint inhibitors [34]. Evidence suggests that chemotherapy, radiotherapy, or DDR inhibitors can produce abnormal nucleic acids that induce tumor immunogenicity independently of neoantigens [35]. Since targeting DNA damage response (DDR) could extend immunotherapy by inducing innate immunity, we speculate that ATM could be a target to enhance the efficacy of immunotherapy in CRC.

ATM inhibitors have been explored in preclinical and clinical studies, but remain widely unknown in colorectal cancer due to the limited number of clinical cases [36–41]. Several clinical trials of ATM inhibitors are ongoing, but only one Phase I trial has concluded, studying the safety and preliminary efficacy of AZD0156 alone or in combination with other anticancer treatments in patients with advanced cancer. Data on the co-inhibition of ATM and immune checkpoints in CRC are still lacking. Whether targeting ATM can be used as an opportunity for immunotherapy of colorectal cancer remains unknown. Therefore, this study aimed to explore the value of ATM as a marker in treating CRC, the anticancer efficacy of ATM inhibitors, and the mechanism of ATM in immune resistance.

## Result

### ATM inhibition activates cGAS/STING pathway in CRC cells and has synergistic effect with radiation

**Fig. 1:**
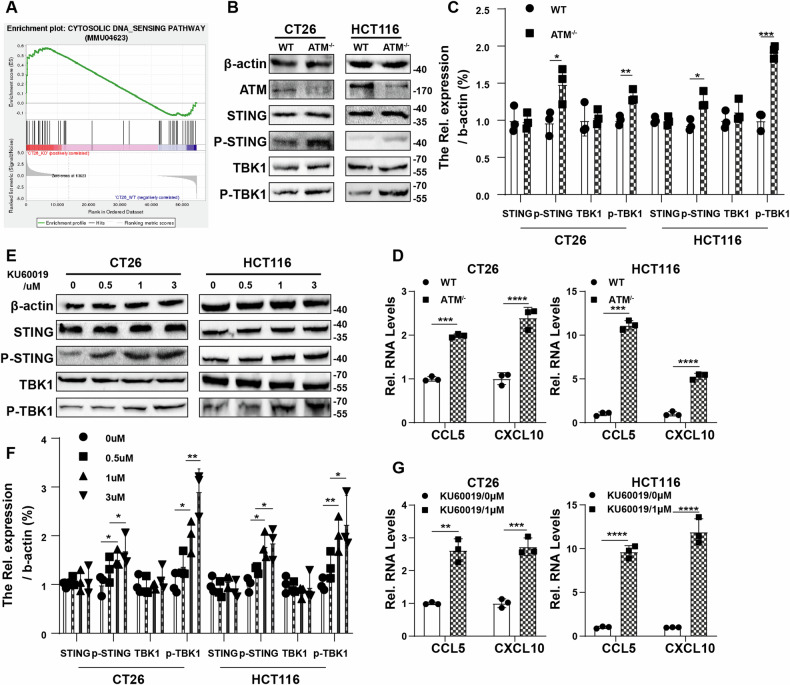
ATM inhibition activates cGAS/STING pathway. **A** Gene Set Enrichment Analysis plot for CYTOSOLIC DNA SENSING PATHWAY. **B**, **C** Immunoblots showing STING, p-STING, TBK1, p-TBK1 and β-actin expression level in vector control and ATM-KO CT26 cells and HCT116 cells. **D** qRT-PCR was used to measure the mRNA level of CCL5, CXCL10 in vector control and ATM-KO CT26 cells and HCT116 cells. **E, F** Immunoblots showing STING, p-STING, TBK1, p-TBK1 and β-actin expression level in CT26 cells and HCT116 cells treated with increasing dose of KU60019 for 24 h. **G** qRT-PCR was used to measure the mRNA level of CCL5, CXCL10 in CT26 cells and HCT116 cells treated with increasing dose of KU60019 for 24 h. * P < 0.05, ** P < 0.01, *** *P* < 0.001, **** *P* < 0.0001.

In this study, we used ATM knockout CT26 cells and ATM knockdown HCT116 cells to investigate the effect of ATM gene deficiency on CRC cells. We performed RNA-seq on vector control CT26 cell line and ATM-KO CT26 cell line to investigate the transcription changes caused by ATM deficiency. Consistent with previous studies [42], ATM-deficient cells showed significant enrichment of genes associated with immune response compared to vector control cells (Fig. S1A–C). In addition, our RNA-seq results showed an enrichment of genes related to the CYTOSOLIC DNA SENSING PATHWAY in ATM-deficient CRC cells (Fig. 1A), suggesting that ATM-deficiency activates cGAS/STING pathway in CRC cells. From our results, ATM-deficient CRC cells showed upregulated expression of cGAS/STING pathway related proteins and upregulated transcription of CXCL10 and CCL5, which are downstream of the cGAS/STING pathway (Fig. 1B–D). Furthermore, we picked KU60019, an ATM inhibitor in clinical trials, to study the impact of drug targeting ATM on CRC cells. We found that KU60019 activates cGAS /STING pathway in CRC cells dose-dependently (Fig. 1E–G). These results suggest that gene deletion or chemical inhibition of ATM could activate the cGAS /STING pathway in CRC cells.

**Fig. 2:**
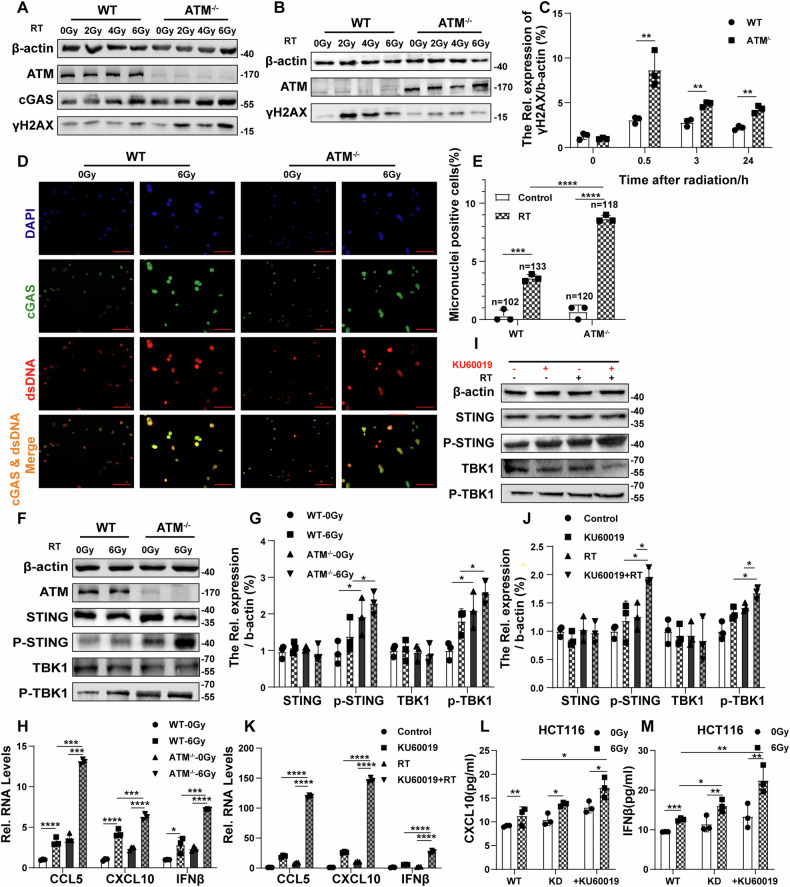
Radiation enhances cGAS/STING pathway activation induced by ATM inhibition. **A** Immunoblots of γH2AX and cGAS in vector control and ATM-KO CT26 cells treated with increasing doses of radiation for 24 h. **B**, **C** Immunoblots of γH2AX in vector control and ATM-KO CT26 cells and its quantitative data analysis at different times after radiation. **D**–**H** Vector control and ATM-KO CT26 cells were irradiated with 6 Gy for 24 h. Fluorescence microscopy was used to assess the dsDNA and cGAS (**D**). Scale bar: 50 μm. The percentage of micronuclei dsDNA positive cells relative to the total number of cells in three randomly selected fields (**E**). Immunoblots showing STING, p-STING, TBK1, p-TBK1, ATM, and β-actin expression levels (**F** and **G**). qRT-PCR was used to measure the mRNA level of CCL5, CXCL10, and IFNβ (**H**). **I–K** CT26 cells were treated with KU60019 (3 μmol/L, 24 h) or radiation (6 Gy, 24 h). Immunoblots showing STING, p-STING, TBK1, p-TBK1, and β-actin expression levels (**I** and **J**). qRT-PCR was used to measure the mRNA level of CCL5, CXCL10, and IFNβ (K). **L**, **M** ELISA was used to detect the level of CXCL10 (**L**) and IFNβ (**M**) in shFluc and shATM HCT116 after different treatments for 24 h. *P < 0.05, **P < 0.01, ***P < 0.001, ****P < 0.0001.

To determine the feasibility of targeting ATM to improve the radiosensitivity of CRC cells, we first performed a series of in vitro experiments. From the results of the colony formation assay and apoptosis assay (Fig. S2A–C), we found that cell growth inhibition and cell death caused by radiation were more severe in ATM-deficient CRC cells. Radiation-induced cell death is not only caused by direct DNA damage but also by the production of reactive oxygen species (ROS), which amplify signals to generate irreversible DNA damage [9, 43]. We further detected ROS production and the expression level of DNA damage marker phosphorylated H2AX (γH2AX) after radiation in ATM-deficient cells [44, 45]. As shown in (Fig. 2A–C and Fig. S2D), ATM-deficient cells produced more ROS and stronger DSB after radiation. Results showed that KU60019 has a synergistic effect with radiation in inhibiting cell proliferation and promoting DNA damage in CRC cells (Fig. S2D–F). Taken together, these results suggest that targeted inhibition of ATM can aggravate DNA damage and cell death caused by radiation.

We know that cGAS is a cytosolic DNA and micronuclei sensor that activates the type I interferon pathway [11–13]. To assess DNA damage in detail, we examined whether cytosolic dsDNA is associated with cGAS. As shown in (Fig. 2D, E), ATM-deficient cells produced more micronuclei after radiation and co-located with cGAS. Western blot analysis showed that the levels of phosphorylated STING and TBK1 were higher in ATM-deficient cells (Fig. 2F, G) with or without radiation. The qPCR results showed that CCL5, CXCL10, and IFN-β were remarkably upregulated after radiation in ATM-deficient cells (Fig. 2H), demonstrating that ATM-deficient cell activates type I interferon pathway after radiation by increasing micronuclei. Besides, KU60019 has a synergistic effect with radiotherapy in activating the cGAS/STING pathway (Fig. 2I–K). Moreover, the Elisa results showed that the upregulation of CXCL10 and IFNβ in the cells was more evident after ATM inhibition combined with radiation (Fig. 2L, M). We concluded that radiation enhances cellular DNA damage and cGAS/STING pathway activation caused by ATM inhibition.

### The MHC class I expression in CRC cells was upregulated by ATM inhibition and augmented by radiation

**Fig. 3:**
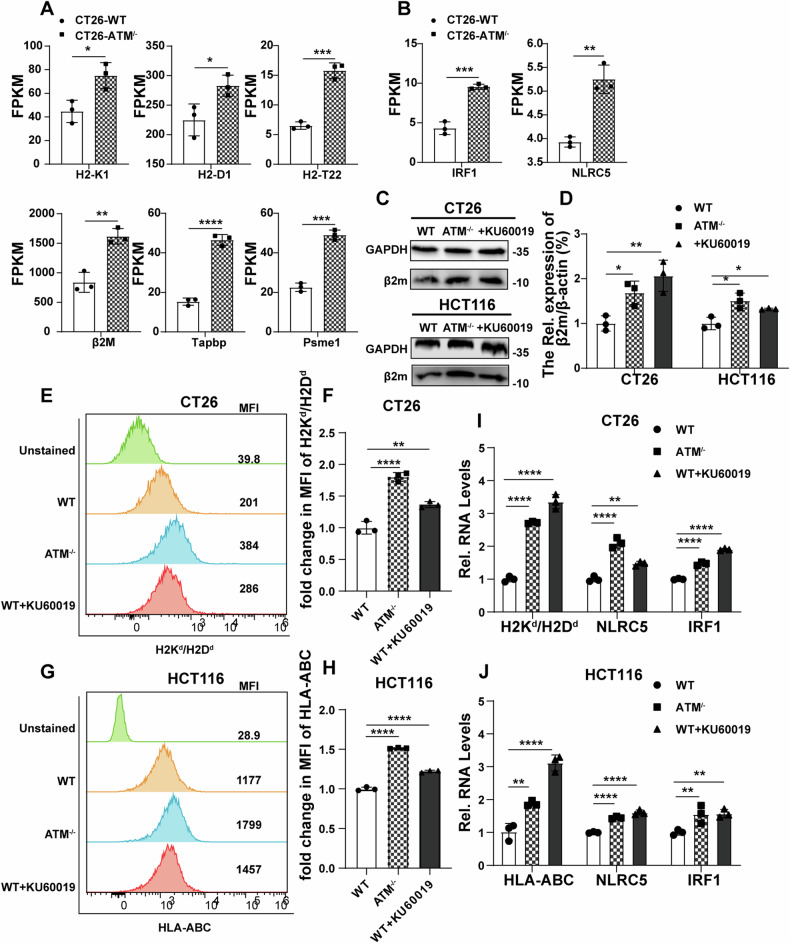
ATM inhibition enhances MHC-I, IRF1 and NLRC5 expression. **A**, **B** FPKM quantification of antigen processing and presentation-related genes (**A**), IRF1, and NLRC5 (**B**). **C**, **D** Immunoblots showing b2M and GAPDH expression levels in vector control, ATM-KO, and KU60019 (3 μM, 24 h) treated CT26 cells and HCT116 cells. **E–J** Vector control, ATM-KO, KU60019 (3 μM, 24 h) treated CT26 cells, and HCT116 cells were immunostained with the anti-MHC-I antibody and analyzed with flow cytometry (**E**–**H**). qRT-PCR was used to measure the mRNA level of MHC-I, IRF1, and NLRC5 (**I** and **J**). *P < 0.05, **P < 0.01, ***P < 0.001, ****P < 0.0001.

From our RNA-seq on vector control and ATM-KO CT26 cell line, we found that multiple immune factors (H2-K1, H2-D1, β2M, IRF1, NLRC5) closely related to MHC class I were upregulated by ATM knockout (Fig. 3A, B), which were verified by qRT-PCR. Western blot and flow cytometry showed that the total b2M protein level and the membrane MHC class I level were upregulated in ATM-deficient cells and KU60019-treated cells (Fig. 3C–H).

At MHC class I promoters, NF-κB interacts with the NF-κB-binding sites of enhancer A, the ISRE binds to IRF family members such as IRF1, and NLRC5 forms an enhanceosome with transcription factors (such as the RFX complex) at the MHC class I promoter to induce MHC class I gene expression [46]. qRT-PCR results showed that inhibition of ATM could promote IRF1 and NLRC5 transcription, and MHC class I upregulation may occur at the transcriptional level (Fig. 3I, J). Moreover, ATM inhibitors induced the transcription of MHC class I and NLRC5 in APCs isolated from mice but inhibited IRF1 transcription (Fig. S3A–C). These results suggest that the mechanism by which ATM inhibition augments the expression of MHC class I may differ between cancer cell lines and APCs, but both involve NLRC5.

To explore whether radiation could amplify the effect of ATM on MHC class I expression, we detected the expression level of MHC class I in the membrane of ATM-deficient and ATM-proficient colorectal cancer cells after radiation and IFN-γ stimulation. After IFN-γ stimulation and radiation, the expression of MHC class I was upregulated in the two cell lines, especially in ATM-deficient tumor cell lines (Fig. S4A–C). The expression level of MHC class II and PD-L1 in ATM-deficient cell lines and ATM-proficient cell lines were upregulated to some extent after IFN-γ stimulation and radiation. Still, the difference was not significant between ATM-proficient cell lines and ATM-deficient cell lines (Fig. S4D–G). Here, we found that ATM inhibition promotes the expression of MHC class I in CRC cell lines after radiation, suggesting that the upregulation of MHC class I after DSBs may be related to ATM in CRC cell lines.

### NF-κB transcriptional pathway is involved in the upregulation of MHC class I, IRF1, and NLRC5 caused by ATM inhibition

**Fig. 4:**
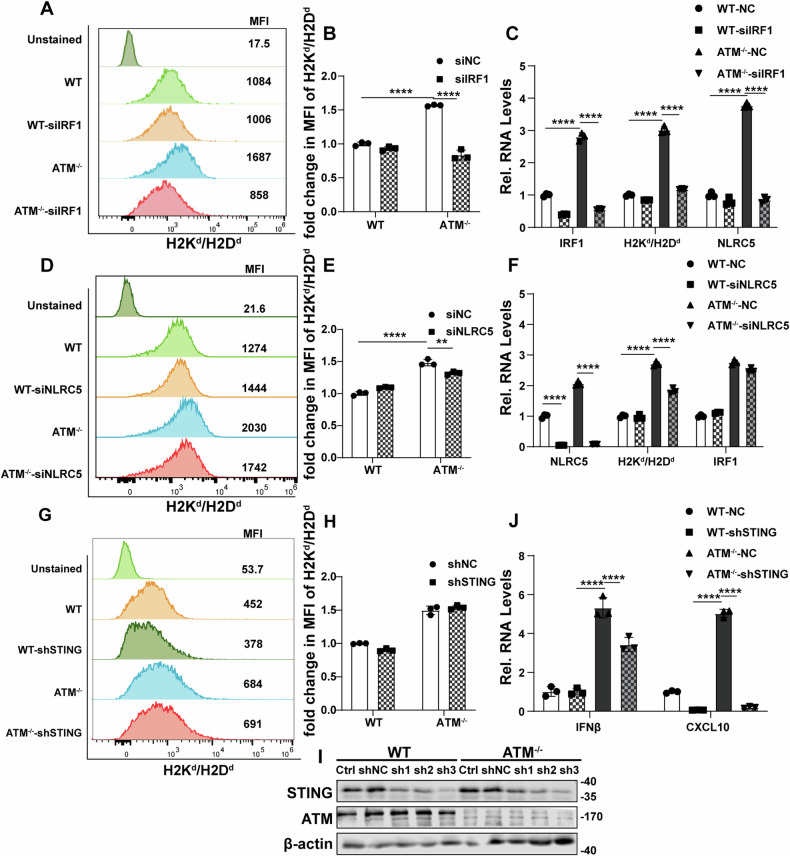
IRF1/NLRC5 is required for ATM inhibition-induced expression of MHC-I. **A–C** Vector control and ATM-KO CT26 cells were transfected with siRNA-Ctrl or siRNA-IRF1 for 48 h. Surface expression of H2Kd/H2Dd was determined by flow cytometry (**A** and **B**). qRT-PCR was used to measure the mRNA level of H2Kd/H2Dd, IRF1 and NLRC5 (**C**). **D–F** Vector control and ATM-KO CT26 cells were transfected with siRNA-Ctrl or siRNA-NLRC5 for 48 h. Surface expression of H2Kd/H2Dd was determined by flow cytometry (**D** and **E**). qRT-PCR was used to measure the mRNA level of H2Kd/H2Dd, IRF1 and NLRC5 (**F**). **G–J** Vector control and ATM-KO CT26 cells were transfected with shRNA-Ctrl or shRNA-STING. Surface expression of H2Kd/H2Dd was determined by flow cytometry (**G** and **H**). STING expression was determined by Immunoblots (**I**). IFNβ and CXCL10 expression were determined by qRT-PCR (**J**). *P < 0.05, **P < 0.01, ***P < 0.001, ****P < 0.0001.

To clarify whether ATM inhibition induces MHC class I expression through increased IRF1 and NLRC5 expression, we used transient transfection with small interfering RNA (siRNA) to knock down IRF1 and NLRC5. We found that IRF1 downregulation not only significantly reduced MHC class I upregulation caused by ATM knockout, but also reduced NLRC5 upregulation caused by ATM knockout (Fig. 4A–C). However, NLRC5 downregulation only reduced ATM deficiency-induced MHC class I upregulation and had no significant effect on IRF1 levels (Fig. 4D–F). These results suggest that ATM inhibition induces the expression of NLRC5 partially through the induction of IRF1. To determine whether STING plays a role in this process, we used short hairpin RNA to knockdown STING (Fig. 4I). We found that STING expression contributes to the upregulation of CXCL10 and IFNβ in ATM-deficient cells, but ATM silencing-induced MHC class I upregulation does not depend on STING expression (Fig. 4G–J).

**Fig. 5:**
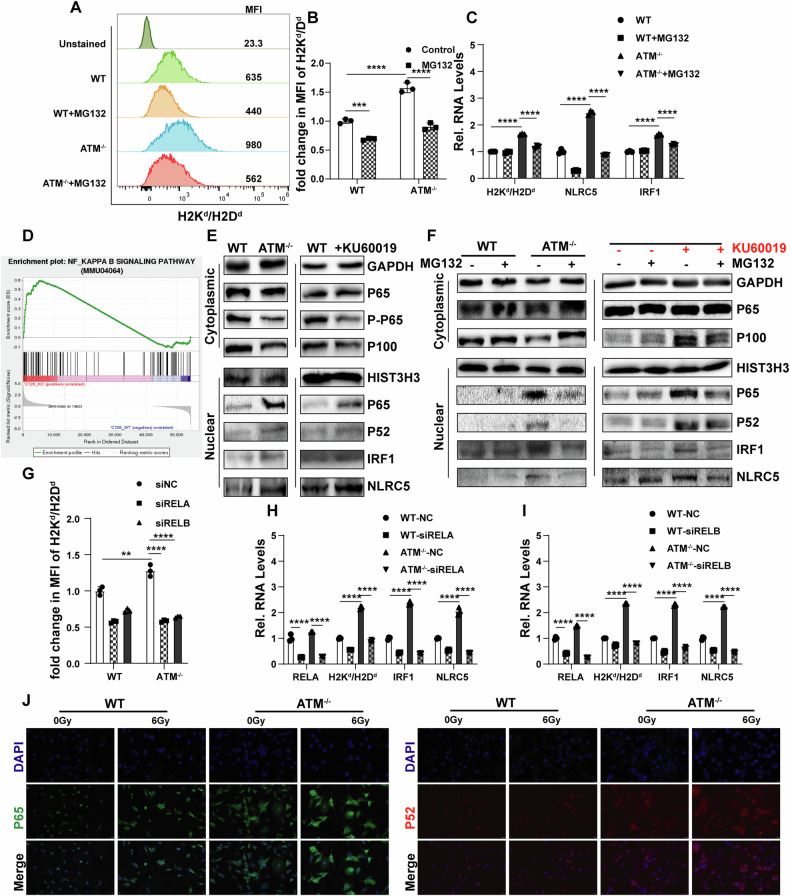
NF-κB transcriptional pathway is involved in the upregulation of MHC-I, IRF1, and NLRC5 caused by ATM inhibition. **A–C** Vector control and ATM-KO CT26 cells were treated with MG132 (8 μM, 1 h). Surface expression of H2Kd/H2Dd was determined by flow cytometry (**A** and **B**). qRT-PCR was used to measure the mRNA level of H2Kd/H2Dd, IRF1 and NLRC5 (**C**). **D** Gene set enrichment analysis plot for NF-KAPPA B SIGNALING PATHWAY. **E**, **F** Representative immunoblot analysis of cytoplasmic and nuclear relocalization of NF-κB family members in Vector control and ATM-KO CT26 cells after different treatments. **G–I** Vector control and ATM-KO CT26 cells were transfected with siRNA-Ctrl, siRNA-RELA, or siRNA-RELB for 48 h. Surface expression of H2Kd/H2Dd was determined by flow cytometry (**G**). qRT-PCR was used to measure the mRNA level of H2Kd/H2Dd, IRF1 and NLRC5 (**H** and **I**). **J** Vector control and ATM-KO CT26 cells were irradiated with 6 Gy for 24 h. Fluorescence microscopy was used to assess P65 and P52. Scale bar: 20 μm. *P < 0.05, **P < 0.01, ***P < 0.001, ****P < 0.0001.

Canonical NF-κB pathway involves activation of the IκB kinase (IKK) complex, leading to phosphorylation of IκB proteins and subsequent ubiquitinylation and degradation by the proteasome. Non-canonical NF-κB pathway involves the NF-κB inducing kinase (NIK) that activates IKKα, thereby leading to the phosphorylation and proteasome-dependent processing of p100. We used MG132, which inhibits proteasome-mediated IκB and P100 degradation processes and NF-κB pathway activation, to determine whether NF-κB plays a role in this process. The results showed that MG132 could reverse the upregulation of MHC class I in ATM-deficient cell lines (Fig. 5A, B). In addition, MG132 suppressed the transcription of IRF1 and NLRC5 in ATM-deficient cell lines (Fig. 5C), indicating the NF-κB pathway is involved in the upregulation of MHC class I, IRF1 and NLRC5 in ATM-deficient cell lines. We next determined whether the canonical and non-canonical NF-κB pathways are activated in basal conditions in the gene deletion or chemical inhibition of ATM. We found a significant enrichment of genes associated with the NF KAPPA B SIGNALING PATHWAY in ATM-deficient CRC cells (Fig. 5D). By isolating cytoplasmic and nucleosomal proteins, we found that basal relocation of P65 and P52 was stronger in ATM-deficient cells and ATM inhibitor-treated cells (Fig. 5E). In addition, we found that inhibition of canonical and non-canonical NF-κB pathways caused by MG132 significantly reversed the up-regulation of IRF1 and NLRC5 (Fig. 5F). Both RELA and RELB knockdown significantly reduced MHC class I expression levels as well as transcription levels of MHC class I, IRF1 and NLRC5 in ATM KO cells (Fig. 5G–I). These results further support our view that MHC class I upregulation induced by ATM inhibition is dependent on activation of NFκB/IRF1/NLRC5 pathway.

We determined that the basic expression level and nuclear relocalization of P65 and P52 in ATM-deficient cells were stronger than in WT cells, and this trend could be amplified by radiation (Fig. 5J). Taken together, these results show that the inhibition of ATM results in NF-κB transcriptional pathway activation, thereby upregulating the expression of IRF1, NLRC5, and MHC class I.

### ATM-deficient tumors have better immunogenicity and response to radiotherapy and immunotherapy in vivo

**Fig. 6:**
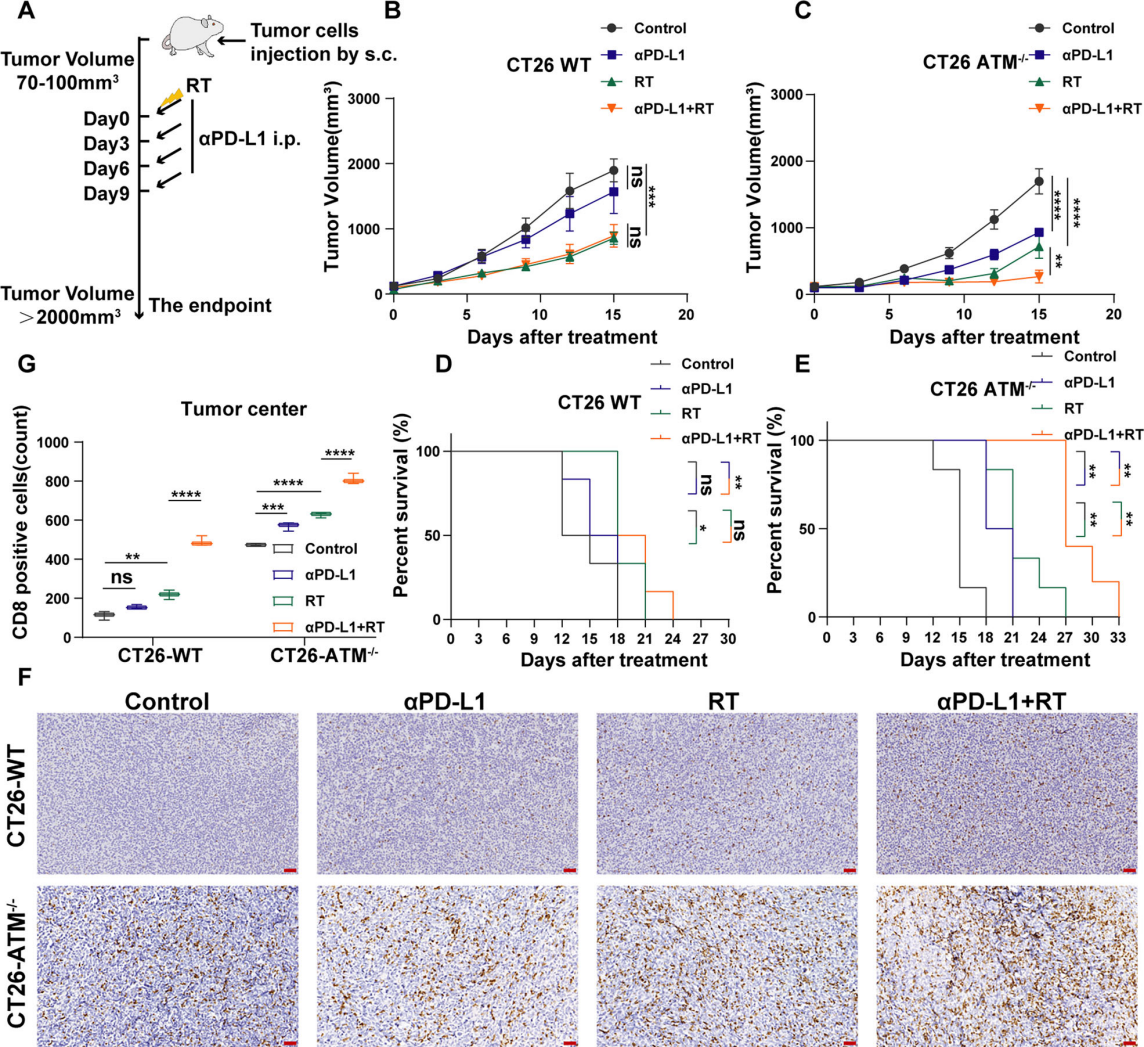
Radiation and anti-PD-L1 enhance therapeutic efficacy in ATM-deficient tumor model. **A** Schema of the mice experimental protocol. **B**, **C** The tumor volume curve of BALB/c mice inoculated with approximately 1 × 10^6^ vector control (**B**) or ATM-KO CT26 cells (**C**). *n* = 6. **D**, **E** Kaplan–Meier survival curves for vector control (**D**) or ATM-KO CT26 tumor (**E**). *n* = 6. **F**, **G** Representative IHC images of CD8 staining (**F**) and statistical analysis (**G**) of vector control and ATM-KO CT26 tumors. Scale bar: 50 μm. *P < 0.05, **P < 0.01, ***P < 0.001, ****P < 0.0001.

BALB/C mice were injected with vector control CT26 cells and ATM-KO CT26 cells to record tumor growth rates after different treatments. We performed in vivo experiments, as shown in Fig. 6A, to observe the difference in efficacy of immunotherapy and radiotherapy between CT26-WT tumors and CT26-ATM^−/−^ tumors. As can be seen from tumor growth curves we recorded, CT26-WT tumors showed a difference only in the combination group (Fig. 6B). However, for CT26-ATM^−/−^ tumors, not only the radiotherapy group but also the anti-PD-L1 group showed significant tumor growth delays, and the combination group had the best efficacy (Fig. 6C). Furthermore, the treatment groups of CT26-ATM^−/−^ tumors showed prolonged overall survival compared to CT26-WT tumors (Fig. 6D, E). Immunohistochemistry was used to determine whether the number of CD8^+^ cells in the tumor center differed in each group. As shown in positive cell count analysis, CT26-ATM^−/−^ tumors had more CD8^+^ cell infiltration in both untreated and treated groups compared with CT26-WT tumors (Fig. 6F, G).

**Fig. 7:**
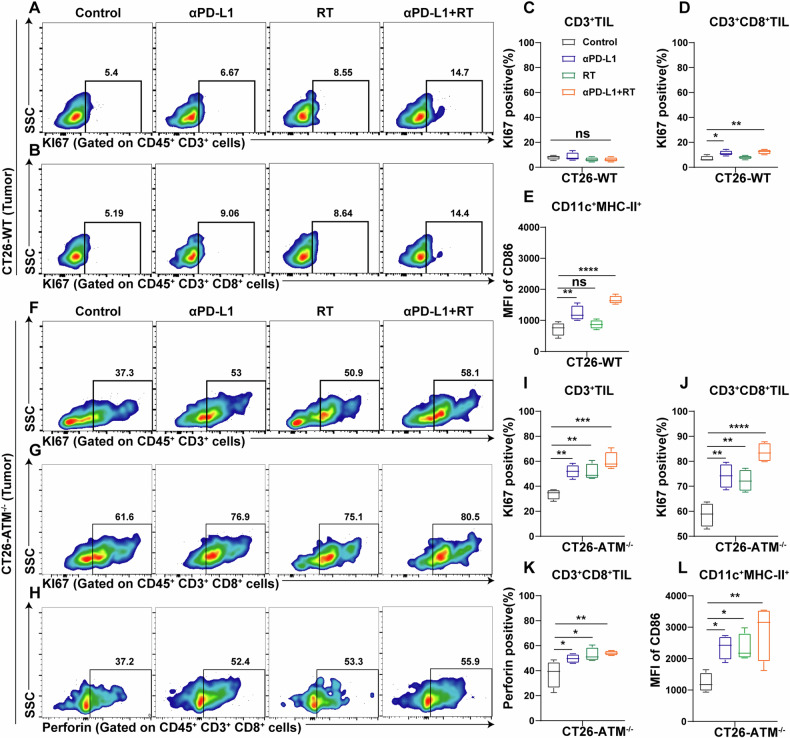
Radiation and anti-PD-L1 enhances lymphocyte function in ATM-deficient tumor model. **A**–**D** Representative flow cytometry profiles of KI67 in CD3+ T cells (**A**), KI67 in CD3+ CD8+ T cells (**B**) from TIL of vector control CT26 tumors and their quantitative analysis (**C** and **D**). *n* = 4. **E** Quantitative analysis of the MFI of CD86 in CD45+ CD11c+ MHC-II+ cells from tumor-draining lymph node of vector control CT26 tumors. *n* = 4. **F–K** Representative flow cytometry profiles of KI67 in CD3+ T cells (**F**), KI67 in CD3+ CD8+ T cells (**J**), and perforin in CD3+ CD8+ T cells (**H**) from TIL of ATM-KO CT26 tumors and their quantitative analysis (**I**, **J** and **K**). *n* = 4. **L** Quantitative analysis of the MFI of CD86 in CD45 + CD11c+ MHC-II+ cells from tumor-draining lymph node of ATM-KO CT26 tumors. *n* = 4. *P < 0.05, **P < 0.01, ***P < 0.001, ****P < 0.0001.

Tumor-infiltrating T cells are the main effector cells in the tumor microenvironment that kill tumor cells. Since activation of CD8 positive T cells depends on MHC class I-TCR stimulation signal, we next explored whether ATM inhibition could further activate tumor-infiltrating T cells. We used flow cytometry to measure Ki67 and perforin to determine the proliferation and cytotoxic ability of tumor-infiltrating T cells (Fig. S5A). Flow cytometry results showed that anti-PD-L1 and radiotherapy both enhanced proliferation ability and cytotoxic ability of tumor-infiltrating T cells in CT26-ATM^−/−^ tumors (Fig. 7F–K), while this effect was not observed in CT26-WT tumors (Fig. 7A–D). In addition to recognizing of antigen-specific signals by TCR, T cell activation also requires CD86/CD80 on APCs to provide costimulatory signals. We analyzed the CD86 expression level of CD11c^+^ MHC class II^+^ cells in tumor-draining lymph nodes to determine the activation of APCs (Fig. S5B). As shown in (Figs. 7E, L and S5C), APCs activation was higher in CT26-ATM^−/−^ tumors than in CT26-WT tumors in all treatment groups.

To test whether ATM-deficient cells were better recognized by CD8+ T cells, we purified mouse CD8+ T cells from draining lymph nodes of CT26 tumors and co-cultured with CT26 WT and ATM-deficient cells. From the results (Fig. S6A, B), CD8+ T cells expressed higher IFN-γ, perforin, and granzyme B after incubated with CT26 ATM-deficient cells compared with CT26 WT cells. These data implied that ATM-KO cells with higher MHC class I expression preferentially enhanced CD8+ T cell activation.

These results suggest that T cells and APCs in ATM-deficient tumors are more functional. ATM-deficient tumors respond better to immunotherapy and radiotherapy compared to WT tumors.

### ATM inhibitor improves the function of lymphocytes in CRC mouse models

**Fig. 8:**
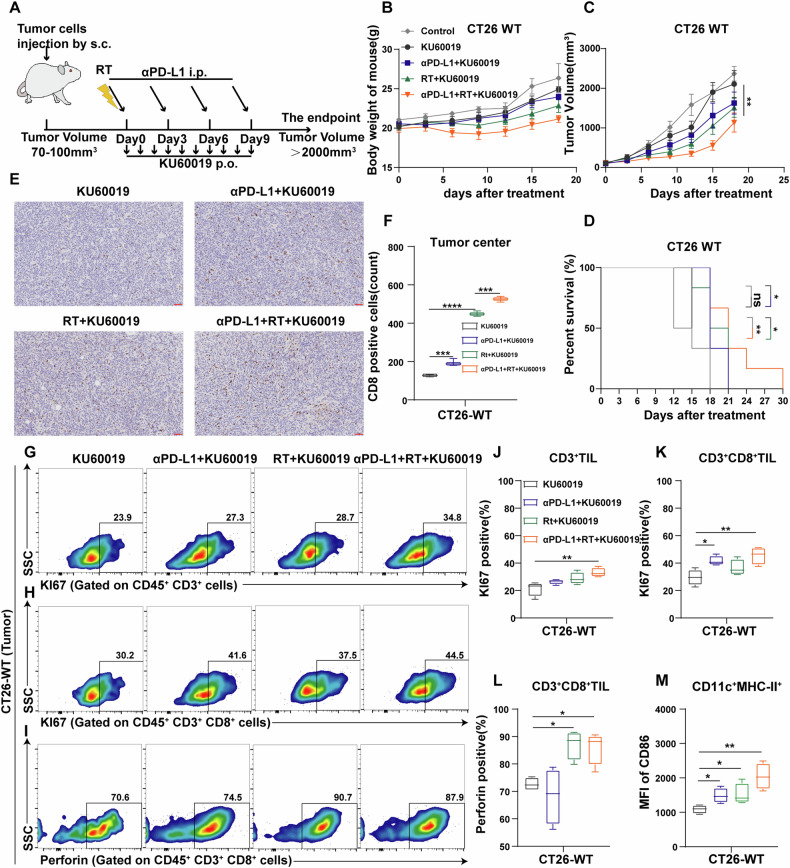
ATM inhibitor is synergistic with radiation and anti-PD-L1 in the syngeneic mouse CRC model. **A** Schema of the mice experimental protocol. **B** Body weight curve of BALB/c mice inoculated with ~1 × 10^6^ CT26 cells and divided into different groups. *n* = 6. **C**, **D** The tumor volume curve (**C**) and Kaplan–Meier survival curves (**D**) of tumor-bearing BALB/c mice. *n* = 6. **E,** **F** Representative IHC images of CD8 staining (**E**) and statistical analysis (**F**) of CT26 tumors. Scale: 50 μm. **G–L** Representative flow cytometry profiles of KI67 in CD3+ T cells (**G**), KI67 in CD3+ CD8+ T cells (**H**) and perforin in CD3+ CD8+ T cells (**I**) from TIL of CT26 tumors and their quantitative analysis (**J**–**L**). *n* = 4. **M** Quantitative analysis of the MFI of CD86 in CD45 +CD11c+ MHC-II+ cells from tumor-draining lymph node of CT26 tumors. *n* = 4. *P < 0.05, ** *P* < 0.01, *** *P* < 0.001, ****P < 0.0001.

Next, we constructed mouse tumor models treated with KU60019 to evaluate the therapeutic effect of ATM inhibitors (Fig. 8A). For CT26-WT tumors, the addition of KU60019 can enhance the efficacy of anti-PD-L1 and radiotherapy to a certain extent (Fig. 8C, D). The body weight of the treated mice did not decrease significantly compared with the untreated mice, indicating that KU60019 was well tolerated in vivo (Fig. 8B).

Besides, KU60019, combined with radiotherapy, significantly increased the number of CD8-positive T cells in the tumor center (Fig. 8E, F). KU60019 combined with anti-PD-L1 or radiotherapy significantly enhanced the function of immune cells of tumor tissues and tumor-draining lymph nodes (Fig. 8G–M). These results suggest that KU60019 has the potential to improve tumor immunogenicity and enhance tumor sensitivity to immunotherapy and radiotherapy.

## Discussion

As a traditional and effective anticancer treatment, radiotherapy not only directly damages tumor cells but also induces DNA damage in tumor cells to activate innate immunity [47, 48]. In addition, radiotherapy is often used as a positive control in studies of the cGAS-STING pathway [49]. However, a number of randomized clinical trials investigating radiotherapy–immunotherapy combinations have failed to demonstrate a therapeutic benefit compared to either modality alone [50, 51], which may be related to the extensive metastasis of patients with advanced tumors and the differences in radiosensitivity due to the heterogeneity of tumors. In this work, we found that ATM inhibition not only enhanced the sensitivity of tumor cells to radiation but also promoted dsDNA and micronucleus production and STING/type I IFN signaling pathway activation in CRC cells, thus increasing innate immune activation. Our data suggest that ATM inhibition can improve radiotherapy efficacy by inducing tumor autonomic type I IFNs response and cell lethal stress in CRC.

In addition, we found significant augment of MHC class I expression and little augment of PD-L1 expression induced by ATM inhibition in CRC cells. We found that ATM inhibition enhanced the expression of MHC class I in CRC cells by activating NF-κB/IRF1/NLRC5 pathway. We found that NLRC5 transcription is partially dependent on IRF1, and both NLRC5 and IRF1 contribute to MHC class I upregulation in ATM-deficient cell lines, which is consistent with previous research that IRF1 regulates NLRC5 transcription by binding to its promoter region [52]. We found that ATM inhibition promotes nuclear relocalization of P65 and P52 in CRC cells, demonstrating that ATM inhibition promotes both canonical and noncanonical NF-κB pathway activation in CRC cells. Inhibition of both canonical and noncanonical NF-κB pathways reversed the upregulation of MHC class I, IRF1 and NLRC5 induced by ATM inhibition. Although previous studies have shown that NF-κB activation depends on ATM activation [53, 54], other studies have found that cells from ATM-knockout mice and individuals with ataxia telangiectasia (AT) exhibit constitutive activation of NF-κB [55–57]. Besides, we found that ATM inhibitor augments MHC class I expression in an NLRC5 dependent and IRF1 independent manner in APCs. Given that both MHC class I and NLRC5 expression could be regulated by NFκB directly [52, 58], the upregulation of MHC class I in BMDM is explicable. We believe that the results of IRF1 downregulation in BMDM may be related to other indirect regulatory functions of ATM. One study found that IRF-1 induction is regulated by ATM-dependent signaling events following exposure to genotoxic stress, but AT fibroblasts (derived from patients with ataxia telagienctasia that are defective in the ATM signaling pathway) was found to had noticeably elevated IRF1 protein levels compared to untransformed NHFs (normal human fibroblasts) [59]. The specific mechanism of IRF1 upregulation induced by DNA damage remain understudied. Given the extensive physiological of the NF-κB and IRF1, we believe that the regulatory role of ATM in deserves further exploration [60, 61].

From animal experiments showed that ATM-deficient tumors and ATM inhibitor-treated tumors respond well to radiotherapy and immunotherapy in the typical CRC p-MMR (mismatch repair gene-proficient) tumor model, CT26 tumor model [62, 63], with the best effect observed in the combination groups. The immunohistochemical and flow cytometry results showed that the immune microenvironment of ATM-deficient tumors was significantly improved compared with wild-type tumors, and the infiltration and function of immune cells were greatly improved. Additionally, although the monotherapy effect of KU60019 was not as significant as ATM gene deletion, it enhanced the therapeutic effects of combined immunotherapy and radiotherapy in the CT26 tumor model. It will be important in future studies to study the anti-tumor effect of ATM inhibitor in autochthonous colorectal tumor model which is closer to the immune environment of human colorectal tumors. Safety data of ATM inhibitors from Phase I clinical trials and preclinical data in combination with other therapies will guide us to translate ATMi therapy in future clinical trials.

**Fig. 9:**
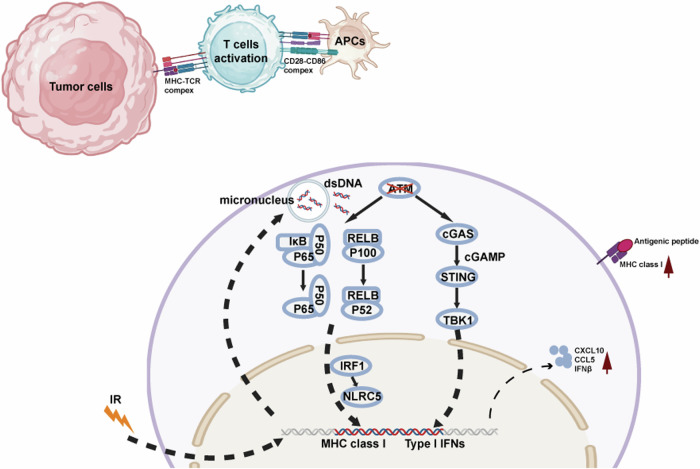
Schematic illustration of ATM inhibition affects STING signaling and MHC Class I expression in tumor cells.

In summary, our study suggested that ATM inhibition could not only stimulate the inflammatory factors through the cGAS/STING pathway but also augment MHC class I expression by activating NF-κB/IRF1/NLRC5 pathway. Radiotherapy could inhibit the growth of tumor cells directly by enhancing cellular stress, and could also amplify the anti-tumor immune effect of ATM inhibition from these two aspects (Fig. 9). Our study supports the therapeutic strategies combining ATM inhibitor with radiotherapy and immunotherapy as well as using ATM mutation as a potential marker of sensitivity to radiotherapy and immunotherapy.

## Materials and methods

### Cell lines and chemicals

Cell lines used in this study were obtained from the Oncology Laboratory, Tongji Hospital, Huazhong University of Science and Technology. CT26 and MC38 were cultured in RPMI-1640 medium (Hyclone, Logan, UT, USA), and HCT116 was cultured in McCoy’s 5A medium (Hyclone, Logan, UT, USA). Cells were cultured in a complete medium containing 10% fetal bovine serum (NEWZERUM) and incubated with 5% CO_2_ at 37 °C. The source of cell lines were verified by STR and tested for mycoplasma contamination.

KU60019 was purchased from Selleck Chemicals (catalog S1570, Houston, TX, USA). Recombinant Human IFN-gamma Protein was purchased from NOVUS (catalog NBP2-34992, CO, USA). Recombinant Mouse IFN-gamma Protein was purchased from R&D Systems (catalog 485-MI, Minneapolis, MN, USA). MG132 was purchased from MCE (catalog HY-13259, NY, USA).

### Animals and animal procedures

The in vivo experiments were performed in standard laboratory conditions in the Tongji Hospital Laboratory Animal Center and approved by the Ethics Committee of Huazhong University of Science and Technology (ID:202112003).

Female BALB/C mice (6–8 weeks, 20–22 g) and male C57BL/6 mice (6–8 weeks, 20–22 g) were bought from Jiangsu GemPharmatech Co., Ltd. (Nanjing, China). 1*10^6^ tumor cells were injected subcutaneously into flanks of BALB/C mice to form tumors. When tumor volume reached 70–100 mm^3^, mice were randomly divided into different groups.

InVivoMab anti-mouse PD-L1 antibody was purchased from BioXcell (clone 10 F.9G2, catalog #BE0101). KU60019 was purchased from Selleck Chemicals (catalog S1570, Houston, TX, USA). Anti-PD-L1 antibody was administered intraperitoneally every 3 days at 10 mg/kg. KU60019 was administered orally at 100 mg/kg once every day. Tumors were treated with radiotherapy on the first day of enrollment. Tumor volume and body weight were measured every three days. The endpoint of the animal’s experiment was tumor volume reaches over 2000 mm^3^.

### Radiotherapy

X-ray irradiator (RS 2000 Biological System irradiator, Rad Source Technologies Inc., USA) was used to irradiate cells and tumor-bearing mice. Cells were irradiated at a dose of 2, 4, and 6 Gy. Mice were anesthetized with 1.5% pentobarbital sodium and received a single fraction of 6 Gy X-ray dose to the area of the subcutaneous tumor.

### Colony-forming assay

Cells seeded in six-well plates at a low density (200–800 cells per well) underwent different treatments and continued to be cultured for 2 weeks to form colonies. Colonies were fixed with 4% buffered paraformaldehyde and stained with 0.01% Crystal violet. Colonies containing 50 or more cells were labeled positive for statistical analysis.

### Detection of ROS

ROS Assay Kit (Beyotime, China) was used to detect intracellular ROS levels. Cells were seeded into six-well plates and incubated overnight. A flow cytometric analysis was performed using CytoFLEX LX (Beckman, Brea, CA, USA). The data were analyzed using FlowJo software v10.

### Apoptosis analysis

Cells were seeded into six-well plates and incubated overnight. After different treatments, FITC Annexin V Apoptosis Detection Kit I (556547, BD Pharmingen™) was used to detect cell apoptotic status. A flow cytometric analysis was performed using CytoFLEX LX (Beckman, Brea, CA, USA). The data were analyzed using FlowJo software v10.

### RT-qPCR analysis

Total cell RNAs were extracted by TRizol reagent (No. 9766, Takara Bio, Shiga, Japan). The RNAs were reversely transcribed to cDNA using an RT reagent kit (Vazyme, Nanjing, China). SYBR Green Master Mix Kit (Vazyme) and RT-PCR system (7900HT, Applied Biosystems, Waltham, MA, USA) were used to detect gene expression levels. The 2^−ΔΔCt^ method was used to determine relative mRNA expressions compared to controls. The primer sequences used are listed in Supplementary Table S1.

### Antibodies and reagents

All antibodies are listed in Supplementary Table S2.

### Western blot analysis

Total cellular protein was extracted using RIPA buffer (Servicebio, China) containing protease and phosphatase inhibitors (Servicebio, Wuhan, China). The extracted proteins were separated by SDS–PAGE and transferred to polyvinylidene fluoride membranes. Membranes were blocked with 5% BSA and incubated with primary antibodies described in Table S2 overnight at 4 °C. Then, the membranes were incubated with secondary antibodies (Promoter, Wuhan, China) and visualized with SuperSignal West Pico Chemiluminescent Substrate (Thermo Scientific, Waltham, MA, USA). Signals on the membrane were analyzed using the G:BOX Chemi X system (Syngene, Cambridge, UK).

### Immunofluorescence (IF)

Cells (10,000–20,000 cells per well) were seeded on 12 mm diameter coverslips in 0.5 ml of culture medium. Cells grown on the coverslips were fixed with 4% paraformaldehyde at room temperature for 20 min and permeabilized using PBS buffer containing 0.05% Tween 20 and 0.005% Triton-X for 10 min. After three washes with PBS, the slides were incubated with PBS containing 1% BSA for 1 h at room temperature to block nonspecific binding. Then, the slides were incubated with different primary antibodies overnight at 4 °C, followed by incubation with fluorescently conjugated secondary antibodies. Finally, all cells were stained with DAPI (Servicebio, Wuhan, China). The cells were visualized under a wide-field fluorescence microscope (Carl Zeiss, BadenWurttemberg, Germany) and analyzed by ImageJ software.

### Enzyme-linked immunosorbent assay (ELISA) analysis

The levels of CXCL10 and IFN-β secreted by the cells in the medium were determined with Human CXCL10/IP10 ELISA Kit (RK00054, ABclonal) and Human interferon Beta ELISA Kit (RK01630, ABclonal) according to the manufacturer’s instruction.

### Cell transfection

The siRNAs targeting IRF1, NLRC5, RELA and RELB were synthesized by RiboBio (Guangzhou, China). Transient transfection was used to deliver the siRNAs. The siRNAs (50 nM) and Lipofectamine 3000 (ABP Biosciences) were gently premixed in a medium without serum according to the manufacturer’s guidelines. The virus shATM and shSTING were purchased from GeneChem (Shanghai, China). Viral particles and polybrene (6 µg/ml, GeneChem) were mixed with a cell culture medium. Puromycin (10 µg/ml, GeneChem) was used to screen cells with successful transfection. Add the mixture to each well-containing cell and medium. Incubate cells at 37 °C in a CO_2_ incubator for 24–96 h until assay for the gene knockdown. The efficacy of the target gene knockdown was verified by qRT-PCR analysis or immunoblotting. The siRNA and shRNA sequences are listed in Supplementary Table S3.

### Flow cytometry

Cultured cells (3*10^5^ cells per sample) were harvested and stained with antibodies against PD-L1, MHC class II and MHC class I at 4 °C for 30 min. A flow cytometric analysis was performed using CytoFLEX LX (Beckman, Brea, CA, USA). The data were analyzed using FlowJo software v10 (BD Biosciences).

Tumor tissues and lymph nodes were harvested from euthanized mice (*n* = 4 per group). Tumor tissues were cut into small pieces and digested with type IV collagenase (Promoter), hyaluronidase, and DNase at 37 °C. The suspensions were filtered using 70 μm nylon cell strainers. eBioscience™ fixable viability dye eFluor™ 780 (65-0865-14, 1:1000; Invitrogen™) was used for live/dead staining, other antibodies used for flow cytometry are listed in Supplementary Table S2. eBioscience™ FOXP3/transcription factor staining buffer set (00-5523-00, Invitrogen™) was used for intracellular staining. A flow cytometric analysis was performed using CytoFLEX LX (Beckman, Brea, CA, USA). The data were analyzed using FlowJo software v10 (BD Biosciences).

### Immunohistochemistry (IHC)

Isolated tumors were fixed with 4% paraformaldehyde at room temperature for 48 h, then dehydrated, and embedded in paraffin wax for further experiments. Various IHC assays were performed according to the manufacturer’s instructions. Anti-CD8α (1:2000, ab217344, Abcam) were used as primary antibodies. Bright-field images were captured using a microscope (EVOS fl auto, Thermo Fisher Scientific, USA).

### RNA-seq assay

The RNA-seq assay was performed by Novogene (Beijing, China). The total RNA of tumor cells (minimum of three samples per group) was extracted using Trizol (Takara Bio) and purified for library preparation and sequencing on an Illumina Hiseq platform. Library preparation, sequencing, and data processing were accomplished by GENEWIZ (South Plainfield, NJ, USA).

### Bone marrow-derived macrophages (BMDMs)

Bone marrow was isolated from C57BL/6 mouse femurs and cultured in RPMI1640 complete media. Cells were incubated at 37 °C and 5% CO_2_. An additional medium with 20 ng/ml GM-CSF (MCE) was added on day 3. Medium containing 20 ng/ml GM-CSF were changed every 3 days. On day 7, the adherent cells were considered BMDMs.

### T cell activation assay

Purified mouse CD8+ T cells were separated in a positive selection procedure using CD8+ T Cell Isolation Kit according to the manufacturer’s instructions (catalog 130-116-478, Miltenyi Biotec) from draining lymph nodes of the tumor. The CD8 + T cell populations were CD45+ CD8+ T cells (>97%). Then, the sorted cells were incubated with CT26 WT and CT26 KO target cells at 5:1 ratio for 72 h. Monensin (BD Bioscience) was added 4 h prior to collecting cells, and subsequently, the cells were collected, intracellularly stained for granzyme B, perforin and IFN-γ and analyzed by flow cytometry. Antibodies used for flow cytometry are listed in Supplementary Table S2.

### Statistical analysis

All statistical analyses were performed by GraphPad Prism 8.0 (San Diego, USA), and the data are presented as mean ± SD unless indicated otherwise. Differences were compared using unpaired Student’s *t*-tests for two groups or one-way ANOVA for multiple groups. The differences were considered statistically significant at *P* < 0.05. Statistical significance was shown as *P < 0.05, **P < 0.01, ***P < 0.001, ****P < 0.0001.

## Supplementary information

Supplementary figure1

Supplementary figure2

Supplementary figure3

Supplementary figure4

Supplementary figure5

Supplementary figure6

Unprocessed WB 1

Unprocessed WB 2

Unprocessed WB 3

Supplementary figure legends

Supplementary tables

## Data Availability

All the data can be obtained by contacting the corresponding author.
