# Correction: ATM inhibition enhance immunotherapy by activating STING signaling and augmenting MHC Class I

**DOI:** 10.1038/s41419-026-08827-6

**Published:** 2026-05-13

**Authors:** Chunya Li, Boyu Wang, Jingyao Tu, Chaofan Liu, Yuan Wang, Junjie Chen, Yongbiao Huang, Bo Liu, Xianglin Yuan

**Affiliations:** 1 Department of Oncology, Tongji Hospital, Tongji Medical College, Huazhong University of Science and Technology, Wuhan, China; 2 Department of Thoracic Surgery, Tongji Hospital, Tongji Medical College, Huazhong University of Science and Technology, Wuhan, China

**Keywords:** Cancer immunotherapy, Cancer microenvironment

Correction to: *Cell Death & Disease* 10.1038/s41419-024-06911-3, published online 20 July 2024

In Fig. 6F, the image of CT26-WT(α-PD-L1 group) was incorrectly used. The following figure should replace the original Fig 6F. This correction does not change the description, interpretation, or the original conclusions of the manuscript.

**Original data** 
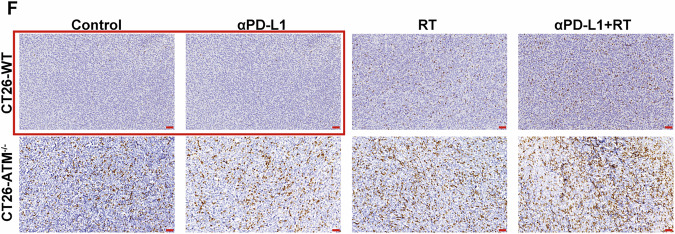


**Amended file** 
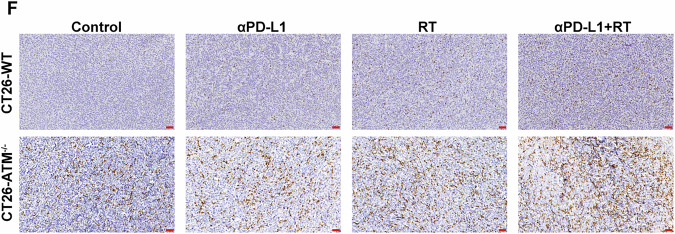


The original article has been corrected.

