# Supplementary figures and images for: ATM inhibition enhance immunotherapy by activating STING signaling and augmenting MHC Class I
Source: Cell Death Dis. 2024 Jul 20;15(7):519. doi: 10.1038/s41419-024-06911-3 (PMC11271473; doi:10.1038/s41419-024-06911-3)

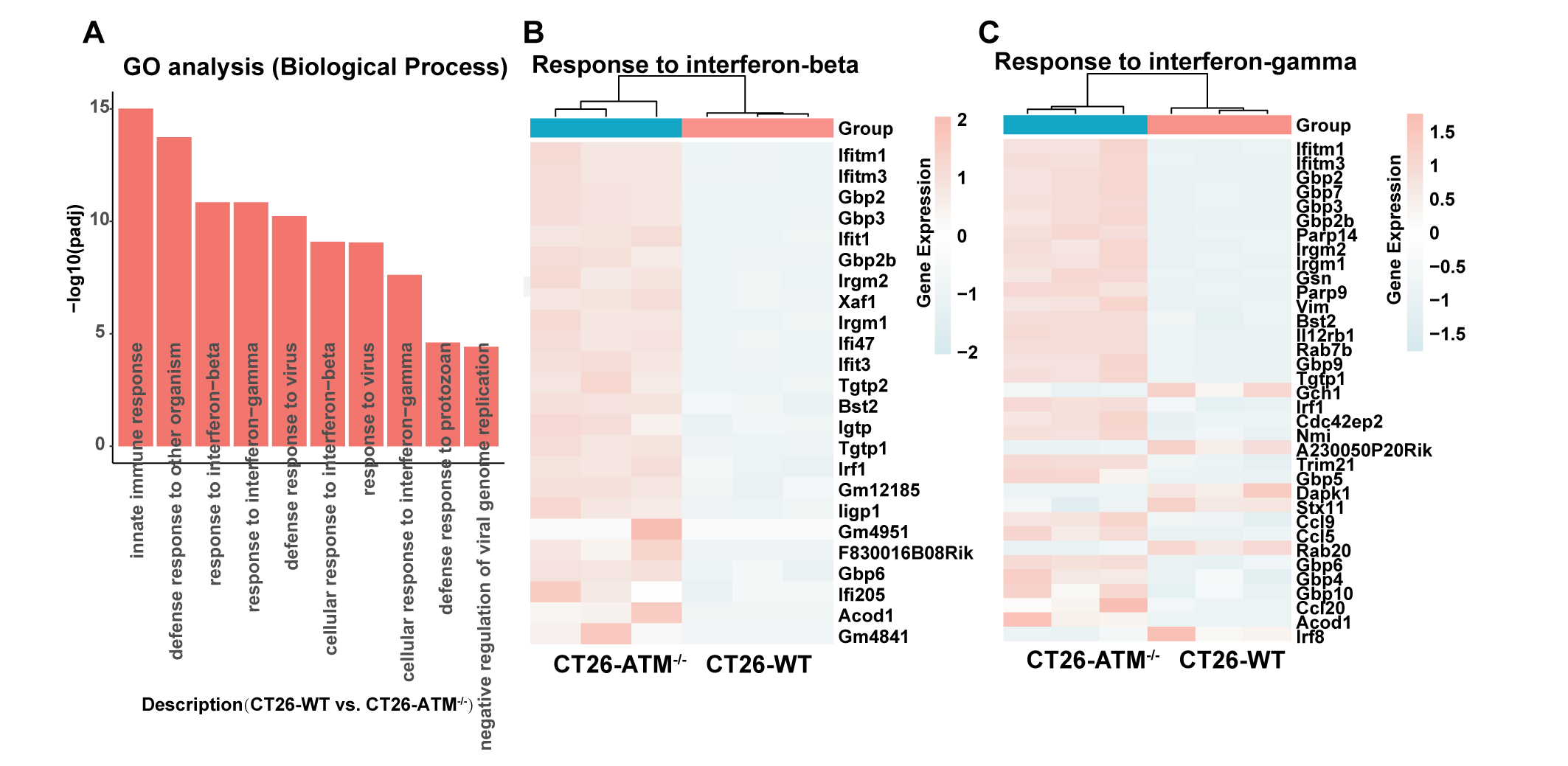

Supplement: Supplementary file 1 — Supplementary figure1 [file 41419_2024_6911_MOESM1_ESM.png]

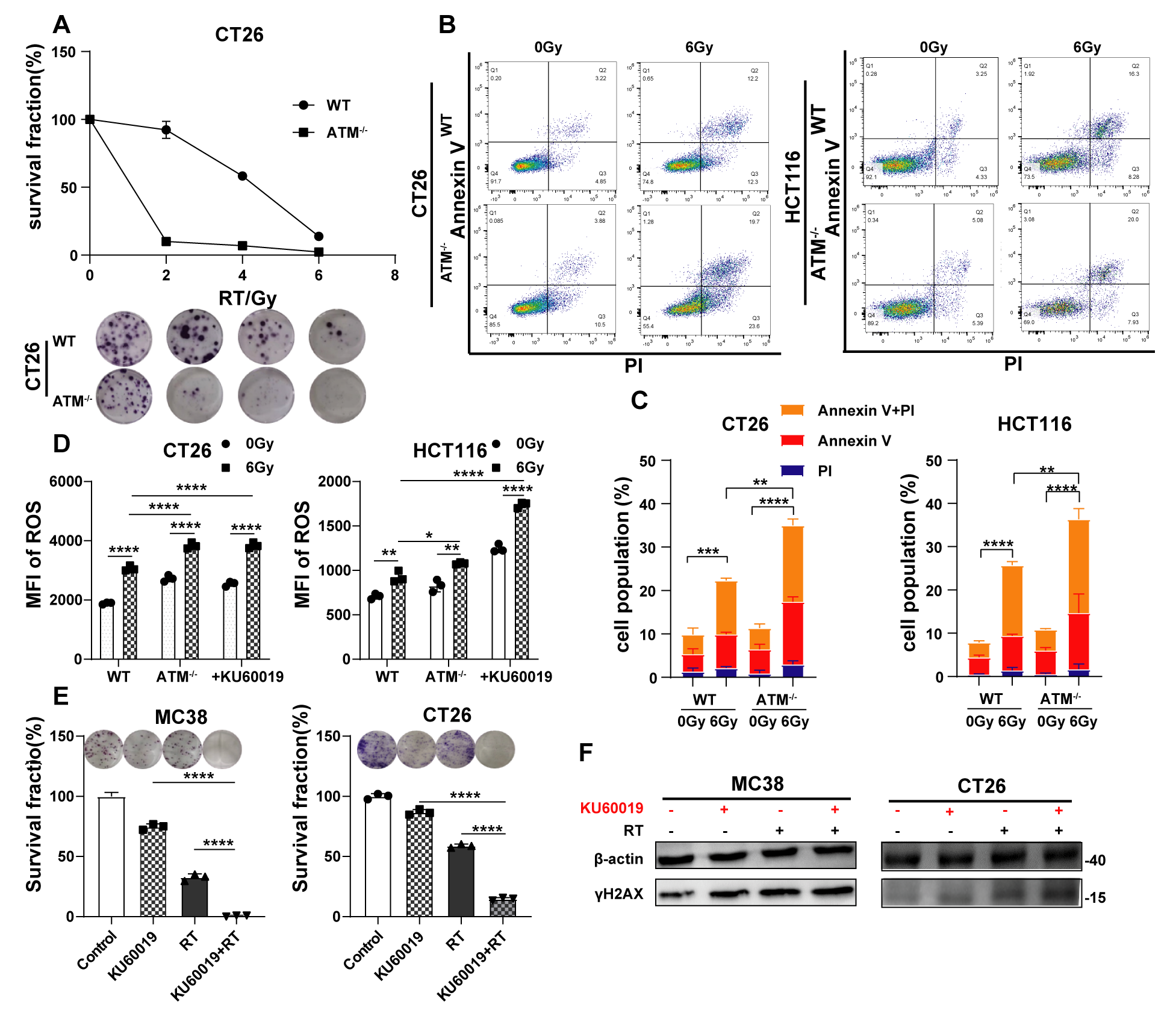

Supplement: Supplementary file 2 — Supplementary figure2 [file 41419_2024_6911_MOESM2_ESM.png]

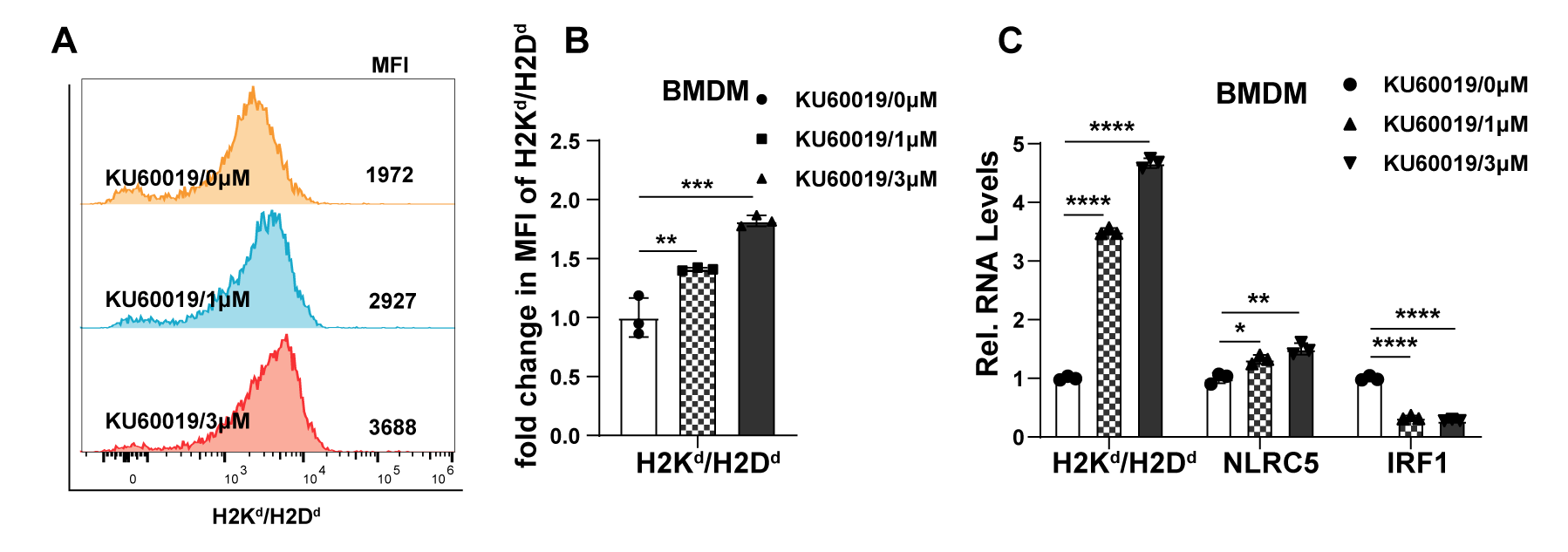

Supplement: Supplementary file 3 — Supplementary figure3 [file 41419_2024_6911_MOESM3_ESM.png]

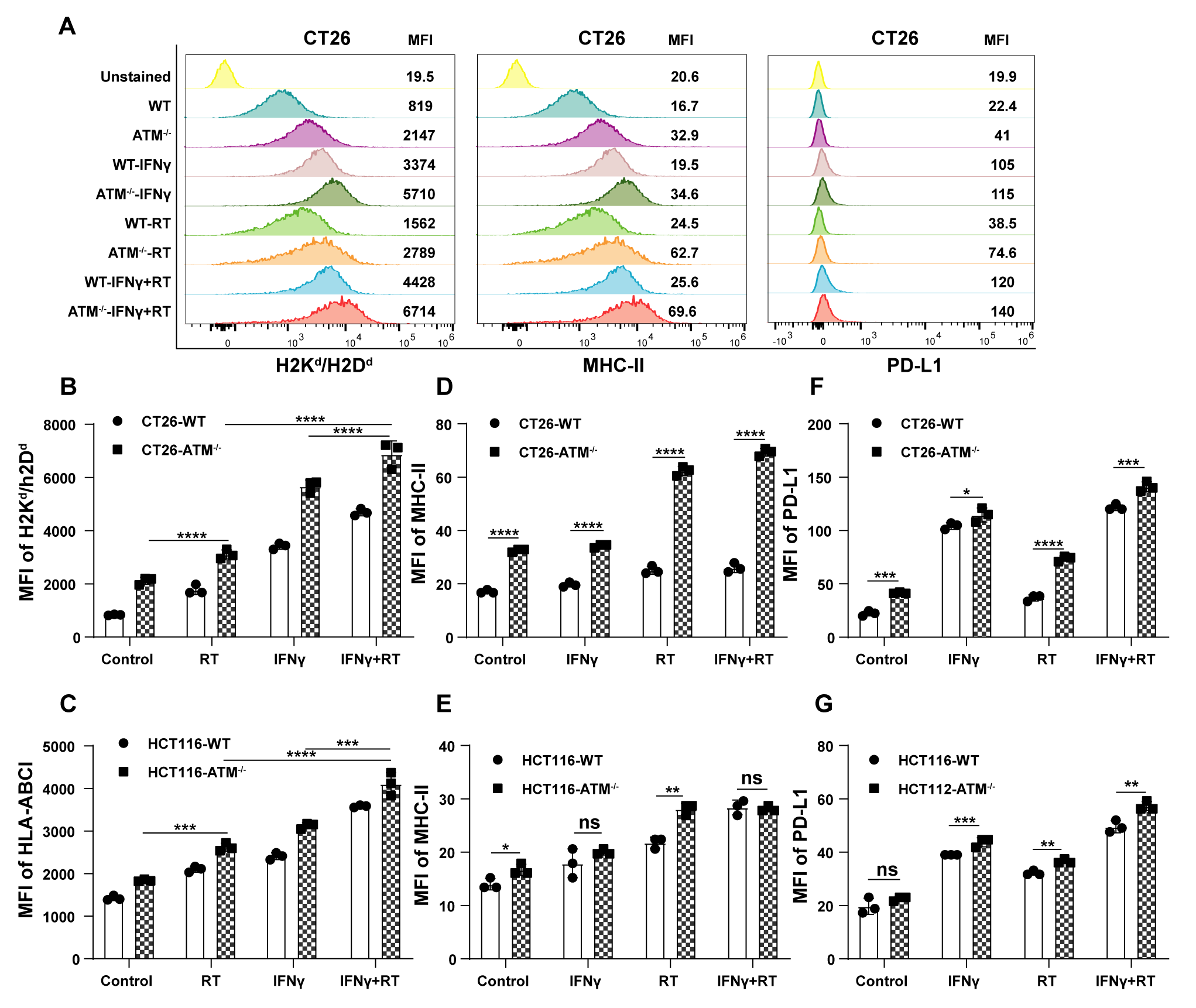

Supplement: Supplementary file 4 — Supplementary figure4 [file 41419_2024_6911_MOESM4_ESM.png]

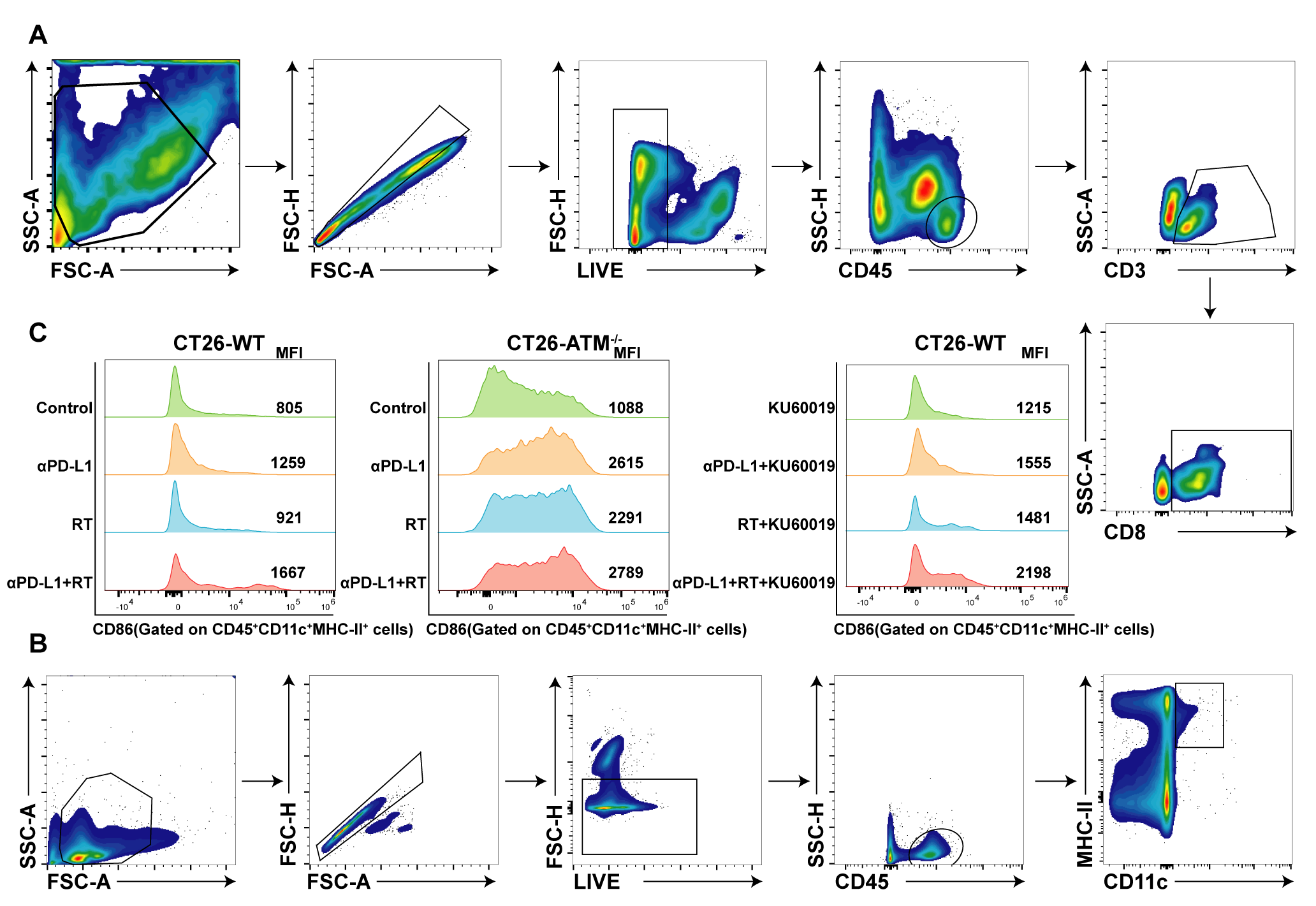

Supplement: Supplementary file 5 — Supplementary figure5 [file 41419_2024_6911_MOESM5_ESM.png]

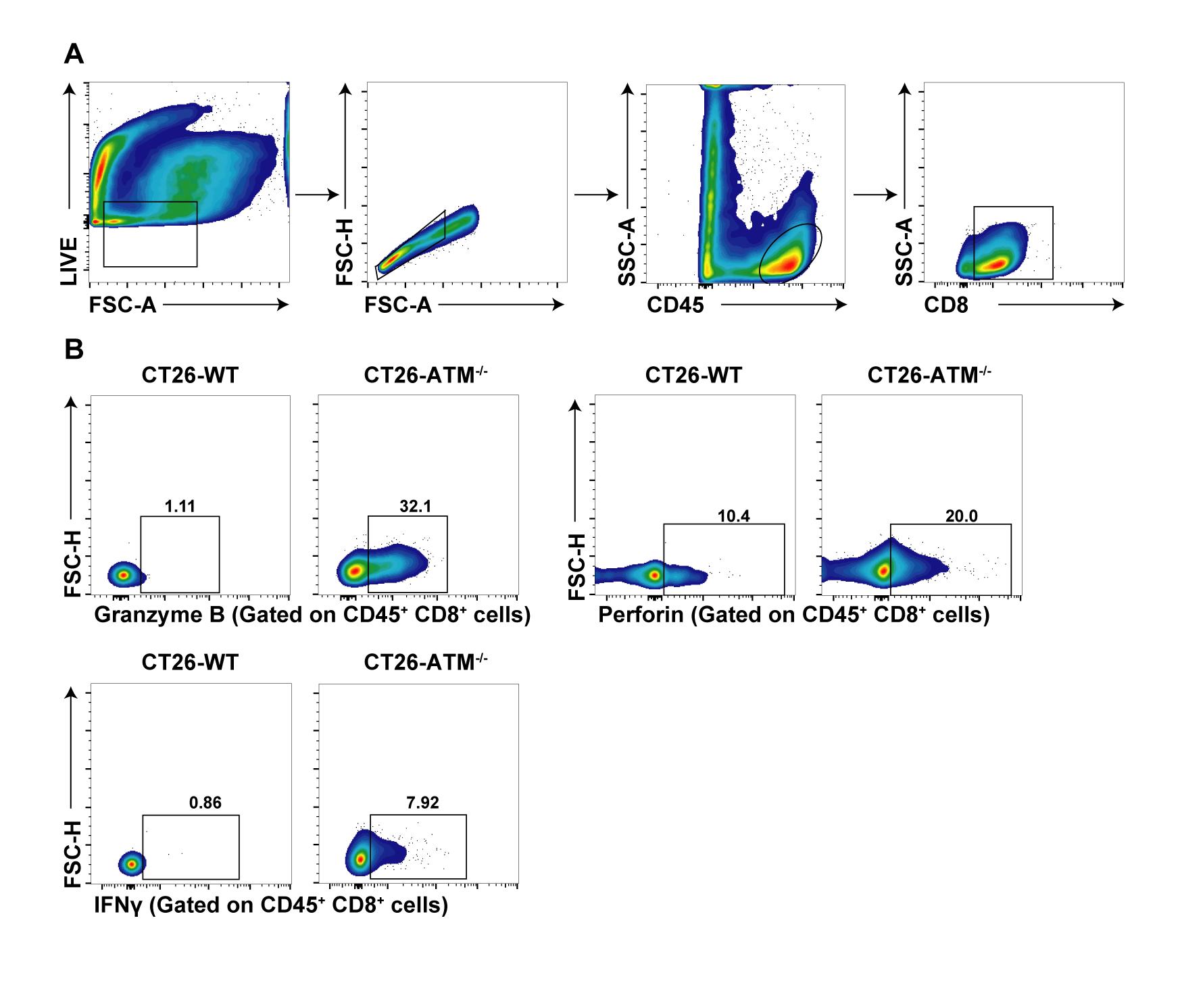

Supplement: Supplementary file 6 — Supplementary figure6 [file 41419_2024_6911_MOESM6_ESM.png]

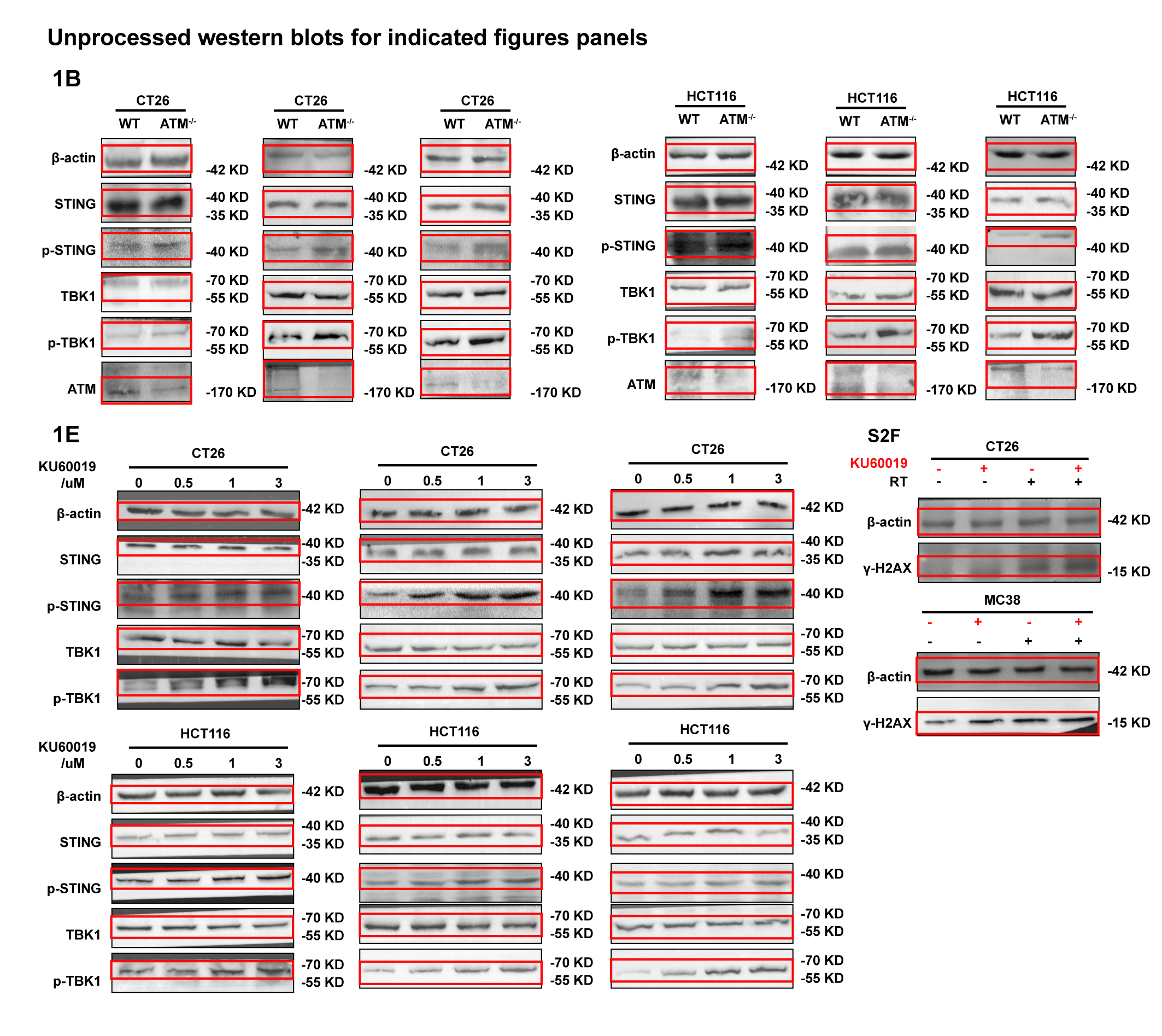

Supplement: Supplementary file 7 — Unprocessed WB 1 [file 41419_2024_6911_MOESM7_ESM.png]

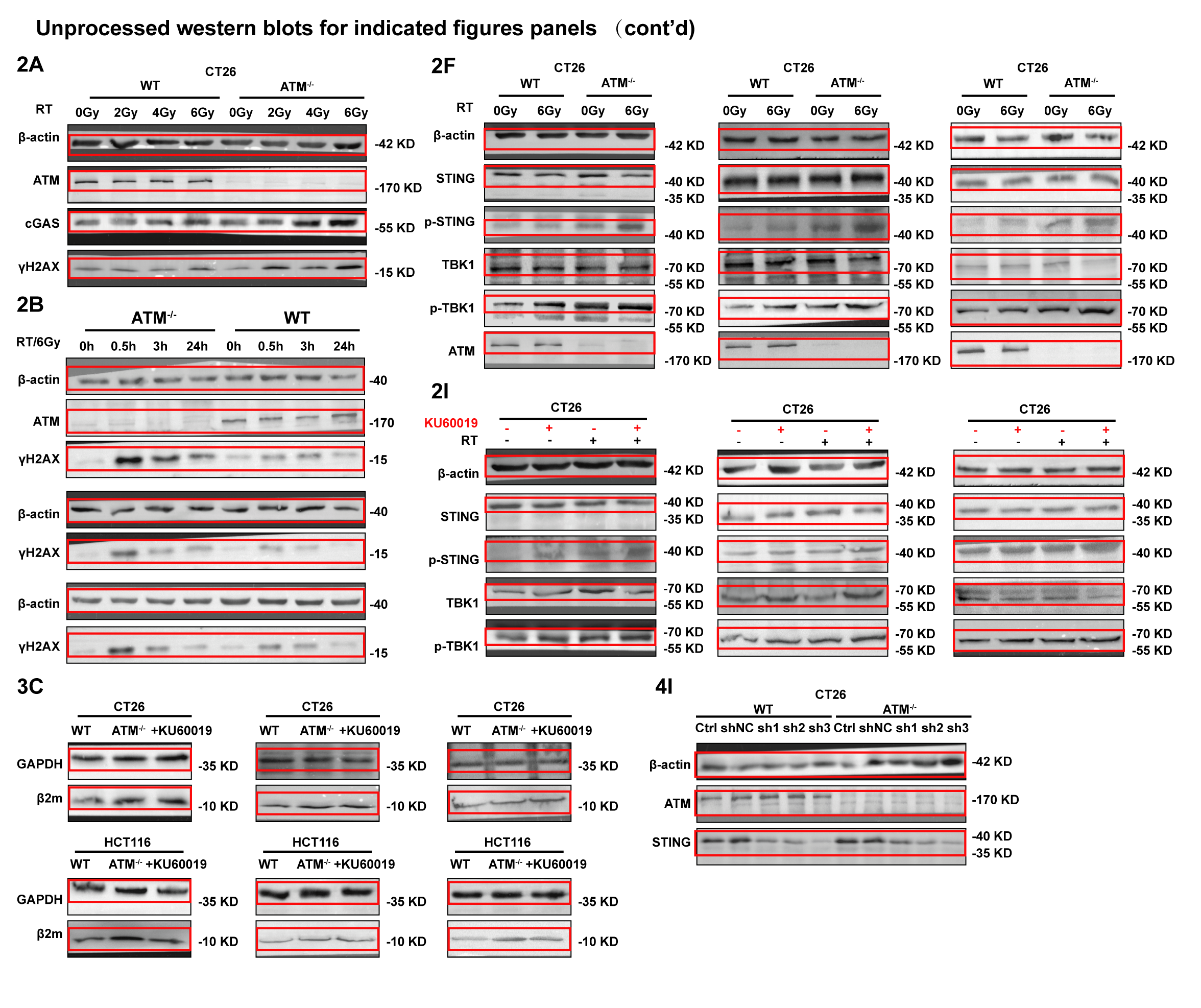

Supplement: Supplementary file 8 — Unprocessed WB 2 [file 41419_2024_6911_MOESM8_ESM.png]

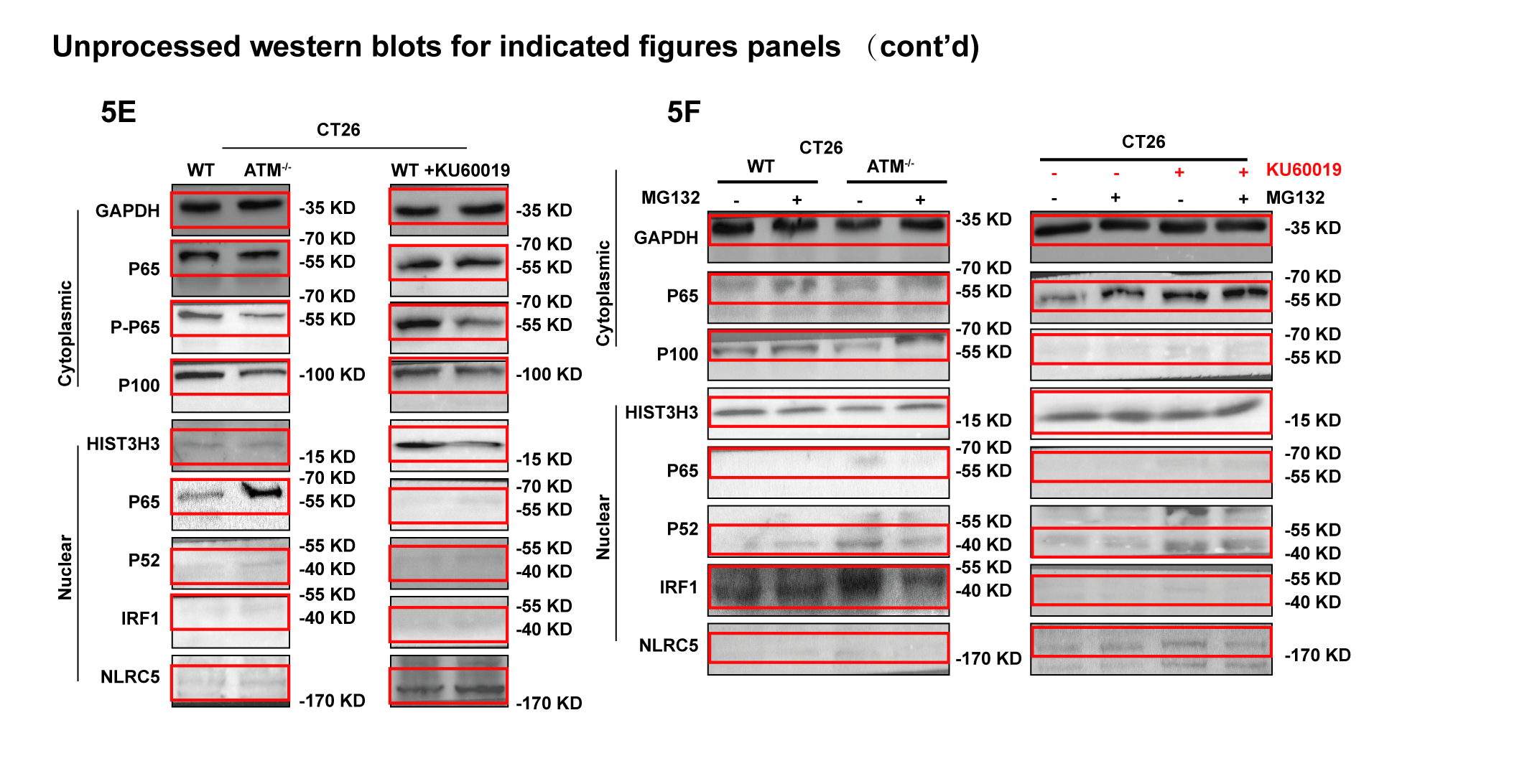

Supplement: Supplementary file 9 — Unprocessed WB 3 [file 41419_2024_6911_MOESM9_ESM.png]
