# Supplementary material for: ATM inhibition enhance immunotherapy by activating STING signaling and augmenting MHC Class I
Source: Cell Death Dis. 2024 Jul 20;15(7):519. doi: 10.1038/s41419-024-06911-3 (PMC11271473; doi:10.1038/s41419-024-06911-3)
Supplement: Supplementary file 10 — Supplementary figure legends [file 41419_2024_6911_MOESM10_ESM.docx]

**Supplementary figure1**

**RNA-seq of vector control and *ATM*-KO CT26 cells**

**(A)** The top 10 significantly enriched GO terms in biological process. **(B and C)** Heat map of response to interferon-beta (B) gene expression and response to interferon-gamma (C) gene expression.

**Supplementary figure2**

**ATM inhibition sensitizes CRC cells to radiation *in* *vitro***

**(A)** Clonogenic survival assays of vector control and *ATM*-KO CT26 cells after a gradient from 0Gy to 6Gy. n=3 wells per group. **(B and C)** Representative flow cytometry profiles of annexin V-FITC/PI staining (B) and statistical analysis (C) of vector control and *ATM*-KO CT26 cells and HCT116 cells after radiation for 24h. **(D)** The reactive oxygen species level of vector control, *ATM*-KO, KU60019 (3μmol/L, 24h) treated CT26 cells and HCT116 cells were detected by flow cytometry after radiation for 24h. **(E)** Clonogenic survival assays of MC38 cells and CT26 cells treated with KU60019 (3μmol/L) and radiation (6Gy). n = 3 wells per group. **(F)** Immunoblots of γH2AX in MC38 cells and CT26 cells treated with KU60019 (3μmol/L, 24h) and radiation (6Gy, 24h). * *P* < 0.05, ** *P* < 0.01, *** *P* < 0.001, **** *P* < 0.0001.

**Supplementary figure3**

**ATM inhibitor enhances the expression of MHC-I in BMDM**

**(A-C)** BMDM from healthy C57BL/6 mouse were incubated with 0-3μM KU60019 for 24h. Surface expression of H2Kd/H2Dd were determined by flow cytometry (A and B). qRT-PCR was used to measure the mRNA level of H2Kd/H2Dd, IRF1 and NLRC5 (C). * *P* < 0.05, ** *P* < 0.01, *** *P* < 0.001, **** *P* < 0.0001.

**Supplementary figure4**

**Effect of ATM silencing on MHC class II and PD-L1 expression in CRC cells**

**(A-G)** Vector control and *ATM*-KO CT26 cells were stimulated with IFNγ (100ng/ml, 24h) and radiation (6Gy, 24h). sh*Fluc* and sh*ATM* HCT116 cells were stimulated with IFNγ (500unit/ml, 24h) and radiation (6Gy, 24h). Surface expression of MHC-I (B and C), MHC-II (D and E) and PD-L1 (F and G) were determined by flow cytometry. * *P* < 0.05, ** *P* < 0.01, *** *P* < 0.001, **** *P* < 0.0001.

**Supplementary figure5**

**Flow cytometry analysis of the immune microenvironment in tumor tissue and tumor draining lymph node**

**(A)** The gating strategy shown for a representative CD45+CD3+CD8+ stained cells excluding debris, dead cells, and doublets in tumor. **(B)** The gating strategy shown for a representative CD45+CD11c+MHC-II+ stained cells excluding debris, dead cells, and doublets in tumor draining lymph node. **(C)** Overlay histograms of fluorescence intensities of CD86 gated on CD45+CD11c+MHC-II+ cells from tumor draining lymph node.

**Supplementary figure6**

**T cell activation assay**

**(A)** The gating strategy shown for a representative CD45+ CD8+ stained cells excluding debris, dead cells, and doublets in tumor. **(B)** Representative flow cytometry profiles of granzyme B, perforin and IFN-γin CD8+ T cells.
