# Supplementary material for: ATM inhibition enhance immunotherapy by activating STING signaling and augmenting MHC Class I
Source: Cell Death Dis. 2024 Jul 20;15(7):519. doi: 10.1038/s41419-024-06911-3 (PMC11271473; doi:10.1038/s41419-024-06911-3)
Supplement: Supplementary file 11 — Supplementary tables [file 41419_2024_6911_MOESM11_ESM.docx]

| **Table S1. The sequences of primers used for experiments in this study.** | | |
| --- | --- | --- |
| **Gene** | **Forward primer (5’-3’)** | **Reverse primer (5’-3’)** |
| β-actin (Mouse) | GAAATCGTGCGTGACATCAAA | TGTAGTTTCATGGATGCCACA |
| CCL5 (Mouse) | CAAGTGCTCCAATCTTGCAGTC | TTCTCTGGGTTGGCACACAC |
| CXCL10 (Mouse) | GTCTGAGTGGGACTCAAGGG | CAACACGTGGGCAGGATAGG |
| IFN-β (Mouse) | CTGGCTTCCATCATGAACAA | AGAGGGCTGTGGTGGAGAA |
| H2K^d^/H2D^d^ (Mouse) | GTTGCAGGTCAGGGTAGCAT | GCACTGCTCTGTCTTCCCTT |
| IRF1 (Mouse) | GGGTCTGAGGTGTAAGGCAG | AGCTCTAGGGCCAGTGCTAT |
| NLRC5 (Mouse) | GCTGGGATGGGTAAGACCAC | ATCATGTTGAGCTGGCGGAA |
| β-actin (Human) | TGACGTGGACATCCGCAAAG | TCTTCATTGTGCTGGGTGCC |
| HLA-ABC (Human) | GCTCAGTGCACGTAAAGTTGAGA | GAGAGACAGCCCACCCTTGT |
| IRF1 (Human) | TGTGGCAAGATCCACACGAA | AGCCTCAGAGGAGCTCTACC |
| NLRC5 (Human) | TAGACTGGGCCTCTCAGACC | ACACAGTCCTCATTGCCCTG |

| **Table S2. List of Antibodies** | | | | | |
| --- | --- | --- | --- | --- | --- |
| **Antibodies** | **Clone** | **Applications** | **Working dilution** | **Supplier** | **Catalog Number** |
| Mouse monoclonal to ds DNA - BSA and Azide free | 35I9 DNA | IF | 1:800 | Abcam | ab27156 |
| Rabbit monoclonal to cGAS | E5V3W | IF | 1:1000 | Cell Signaling Technology | 79978 |
| Rabbit monoclonal to ATM | EP1890Y | WB | 1:5000 | Abcam | ab81292 |
| Rabbit monoclonal to cGAS | D3O8O | WB | 1:1000 | Cell Signaling Technology | 31659 |
| Rabbit monoclonal to γ-H2AX | 20E3 | WB | 1:1000 | Cell Signaling Technology | 9718 |
| Rabbit monoclonal to STING | D1V5L | WB | 1:1000 | Cell Signaling Technology | 50494 |
| Rabbit monoclonal to pSTING | D8F4W | WB | 1:1000 | Cell Signaling Technology | 72971 |
| Rabbit monoclonal to TBK1 | D1B4 | WB | 1:1000 | Cell Signaling Technology | 3504 |
| Rabbit monoclonal to pTBK1 | D52C2 | WB | 1:1000 | Cell Signaling Technology | 5483 |
| Rabbit monoclonal to β2m | D8P1H | WB | 1:1000 | Cell Signaling Technology | 12851 |
| Rabbit monoclonal to NF-κB P65 | D14E12 | WB, IF | 1:1000 | Cell Signaling Technology | 8242 |
| Rabbit monoclonal to Phospho-NF-κB p65 (Ser536) | 93H1 | WB | 1:1000 | Cell Signaling Technology | 3033 |
| Rabbit monoclonal to NF-κB2 P100/P52 | D7A9K | WB, IF | 1:1000 | Cell Signaling Technology | 37359 |
| Alexa Fluor® 488 anti-human HLA-A,B,C | W6/32 | FC | 1:100 | BioLegend | 311415 |
| PE anti-human CD274 (B7-H1, PD-L1) | 29E.2A3 | FC | 1:100 | BioLegend | 329706 |
| PE anti-human HLA-DR | LN3 | FC | 1:100 | BioLegend | 327007 |
| PE anti-mouse H2Kd | SF1-1.1 | FC | 1:100 | BioLegend | 116607 |
| APC anti-mouse CD274 (B7-H1, PD-L1) | 10F.9G2 | FC | 1:100 | BioLegend | 124311 |
| Brilliant Violet 510^TM^ anti-mouse CD45 | 30-F11 | FC | 1:100 | BioLegend | 103138 |
| FITC anti-mouse CD3 | 17A2 | FC | 1:100 | BioLegend | 100204 |
| PE anti-mouse CD8a | 53-6.7 | FC | 1:100 | BioLegend | 100708 |
| Brilliant Violet 421^TM^ anti-mouse CD4 | RM4-4 | FC | 1:100 | BioLegend | 116023 |
| PE/Cyanine7 anti-mouse KI-67 | 16A8 | FC | 1:100 | BioLegend | 652425 |
| APC anti-mouse Perforin | S16009A | FC | 1:100 | BioLegend | 154304 |
| PE anti-mouse I-A/I-E | M5/114.15.2 | FC | 1:100 | BioLegend | 107607 |
| APC anti-mouse CD11c | N418 | FC | 1:100 | BioLegend | 117310 |
| PE/Cyanine7 anti-mouse CD86 | GL-1 | FC | 1:100 | BioLegend | 105013 |
| PE anti-mouse IFN-γ | XMG1.2 | FC | 1:100 | BioLegend | 505807 |
| FITC anti-human/mouse Granzyme B | GB11 | FC | 1:100 | BioLegend | 515403 |

| **Table S3. Cell transfection** | |
| --- | --- |
| Target | Sequence (5’-3’) |
| Mouse-*Atm*-sgRNA | AAACAGTTCAACATTTAGAT-AGG |
| Human-ATM-shRNA | GCCTCCAATTCTTCACAGTAA |
| Mouse*-Sting*-shRNA | CAACATTCGATTCCGAGATAT |
| Mouse-*Irf1*-siRNA | GGACATTGGGATAGGCATA |
| Mouse-*Nlrc5*-siRNA | GAGTCTGCAGAGCCAGGAA |
| Mouse-*Rela*-siRNA | UUGCGCUUCUCUUCAAUCCTT |
| Mouse-*Relb*-siRNA | UUGCACCUUGUCACAGAGCTT |
